# Maternal high sugar and fat diet benefits offspring brain function via targeting on the gut–brain axis

**DOI:** 10.18632/aging.202787

**Published:** 2021-03-26

**Authors:** Dongdong Wang, Haiting Zhang, Miao Zeng, Xiaocui Tang, Xiangxiang Zhu, Yinrui Guo, Longkai Qi, Yizhen Xie, Mei Zhang, Diling Chen

**Affiliations:** 1 State Key Laboratory of Applied Microbiology Southern China, Guangdong Provincial Key Laboratory of Microbial Safety and Health, Guangdong Institute of Microbiology, Guangdong Academy of Sciences, Guangzhou 510070, Guangdong, China; 2 Guangdong Second Provincial General Hospital, Guangzhou 510000, Guangdong, China; 3 Chengdu University of Traditional Chinese Medicine, Chengdu 610075, Sichun, China; 4 Academy of Life Sciences, Jinan University, Guangzhou 510000, Guangdong, China

**Keywords:** maternal diet, pregnancy nutrition, gut-brain axis, cholinergic neurons, GABAergic neurons

## Abstract

A recent study showed that a gestational high fat diet protects 3xTg-AD offspring from memory impairments, synaptic dysfunction, and brain pathology. However, it is unknown whether this diet exerts the same effects on normal mice or on other functions, and if so, how. In the present study, mother mice were pre-fed a high sugar and high fat (HSHF) diet for 1 month and then fertilized; the HSHF diet was continued until birth and then mother mice were returned to a standard diet. The gut microbiota, and intestinal and brain functions of the offspring were dynamically monitored at 7, 14, 28, and 56 days old until 16 months of age. Results showed that the HSHF diet significantly affected the gut microbiota structure of the offspring, especially during the early life stage. In addition, in the HSHF diet offspring, there were influenced on various types of neurons, including cholinergic and GABAergic neurons, on autophagy levels in the brain, and on inflammation levels in the intestinal tract. When the offspring grew older (16 months), we found that some genes of benefit against nervous system disease were activated, such as *Lhx8*, *GPR88*, *RGS9*, *CD4*, *DRD2*, *RXRG*, and *Syt6*, and the expression of cholinergic and GABAergic neurons biomarker protein increased. Although the inflammation levels in the nervous and peripheral systems showed no obvious differences, the AFP level of individuals on the HSHF diet was much higher than those on the standard diet, suggesting that more accurate and/or personalized nutrition is needed. Taken together, the results show that a maternal HSHF diet benefits the offspring by reducing the risk of nervous diseases, which might depend on *LHX8* activation to modulate cholinergic and GABAergic neurons via the gut–brain axis, but still need much more deep studies.

## INTRODUCTION

A hot topic of research in recent years, the gut microbiota and/or its metabolites have been shown by an increasing amount of experimental data to be vital to our health. The gut microbiota can help to extract energy from the diet, synthesize vitamins, promote immune system maturation, and maintain the blood–brain barrier [1–3]. The microorganisms of the gut play different roles and show a specific division of labor [1]. A forward chemical genetic screen revealed that gut microbiota metabolites can potentially modulate nearly all aspects of host physiology [1]. The development of the gut microbiota in infants can be divided into three distinct stages: (i) during the development period, *Bifidobacterium* occupies a dominant position; (ii) during the transition period, the types of microorganisms in the gut microbiota begin to become diversified; (iii) during the stable period, the variation in bacterial species is minor [2]. A HSHF diet or an unbalanced diet with insufficient dietary fiber causes gut microbiota imbalance and reduces the number of bacteria [4]. Furthermore, a HSHF diet during pregnancy also affects the healthy development of future generations [4, 5]. Therefore, we should pay more attention to the transmission of gut microbiota from ancestor to offspring.

Mothers are the main source of infant gut microbiota involved in early colonization. One study found that the microbiota of wild rats is distributed within 10 generations, and that most of the microorganisms in the microbiota of the progeny come from vertical transmission via the parents; the probability of horizontal transmission is low [6]. However, heredity is not the main reason for the colonization and development of the infant gut microbiota. The environment is the major factor affecting the composition, abundance, and distribution of the gut microbiota in infants [7], with common environmental factors including delivery methods, feeding methods, and external contact [2, 8]. During a healthy pregnancy, studies have found bacteria in the uterus, placenta, and amniotic fluid; therefore, it is speculated that the offspring will begin to contact the microbiota in the uterus, and that this may begin the initial colonization of intestinal microbiota [9, 10]; however, this idea is currently controversial. In short, in the first few years of life, an infant gradually forms its own stable microbial community ecology through continuous exposure to new environments [11]. When the gut microbiota of a pregnant woman is disturbed, the gut microbiota of the infant will be affected accordingly. For example, pregnant women with abnormal gut microbiota who suffer from gestational diabetes mellitus [12], inflammatory bowel disease [13], or obesity [14] have offspring in whom the colonization and formation of early gut microbiota are affected. A number of experiments have shown that antibiotics can interfere with the gut microbiota of pregnant women during pregnancy, thereby affecting the flora and immunity of their offspring [15]. Supplementation with probiotics during pregnancy can increase the gut microbiota of pregnant women and change the development of the flora in their offspring [16]. Therefore, in order to harmonize the functioning of the intestinal system, it is very important for pregnant women to maintain their gut microbiota in a stable state; that is, it is very important for pregnant women to maintain a healthy gut microbiota.

When the gut microbiota of a pregnant woman is disturbed, immune system development in the offspring is compromised [17]. In mice, as the offspring grow up and age, the inflammatory level of the body continues to rise. Microglia, which are the immune cells of the central nervous system, are activated during aging or neurodegenerative diseases. The gut microbiota is one of the important factors affected by age-related inflammation, and this accelerates cognitive decline [18, 19]. A basic level of autophagy is also usually required for normal life. Autophagy can be induced through the mammalian target of rapamycin (mTOR) pathway in response to external nutritional deficiencies. Fernández et al. found that the *beclin-1* mutation works by releasing *beclin-1* from negative regulators, and inferred that mammals can prevent premature aging by increasing cell autophagy, thereby achieving a prolonging of their lifespan [20]. Therefore, inflammation and autophagy are important indicators in offspring from birth to old age.

The gut-brain axis, which could be interpreted as a two-way signal communication network between the gut and the brain, consists of hormonal and neural signal loop [1, 21]. Millions of nerve cells, highly specialized differentiated cells and microbes in the digestive tract, which can communicate with our brain cells to regulate the important physiological and behavioral activities. And previous studies showed that a variety of diseases linked to the communication defects of gut-brain, including metabolic disorders [22], gastrointestinal disorders [12], and central nervous system disorders as mood [23, 24], cognition, autism, Alzheimer's disease (AD) [25] and Parkinson's disease [26]. Thus, the gut or gut microbiota may play important roles in the brain development.

The LIM homeobox protein 8 (*Lhx8*) can promote the development and differentiation of specific neurons in specific directions. *Lhx8*-specific expression in the medial ganglionic eminence (MGE) plays an important role in the development and differentiation of the nervous system, especially of cholinergic neurons [27, 28]. Therefore, *Lhx8* has attracted more and more attention with respect to the mechanisms underlying the formation, differentiation, regeneration, and regulation of neurons. Striatal neurons in the intermediate zone include cholinergic neurons and GABA (γ-aminobutyric acid)-ergic neurons, which project fibers into the hippocampus and cerebral cortex. A large number of studies have proved that these neurons are closely associated with cognitive learning ability. When the expression of *Lhx8* decreases, the development of cholinergic neurons and GABAergic neurons increases [27–29]. The onset of AD is related to cortical cholinergic neuron transmitter dysfunction, inadequate acetylcholine synthesis, decreased levels of non-cholinergic transmitters such as 5-hydroxytryptamine (5-HT) and GABA, and apoptosis [30, 31]. The onset of schizophrenia is related to the regulation of the glutamate–glutamine–GABA cycle by the gut microbiota [32]. The gut microbiota plays an important role in neurodegenerative diseases; therefore, pregnant women with a HSHF diet can modulate cholinergic and GABAergic neurons via the intestinal–brain axis by activating LHX8, thereby affecting the brain function of the next generation.

A previous study demonstrated that hormone loss induced by a dietary change increases central adiposity, and promotes AD development [33]. It has also been reported that a high-fat diet (HFD) accelerates cognitive impairment by enhancing oxidative stress and aggravating neuronal apoptosis via inactivation of the Nrf2 (Nuclear factor, erythroid 2 like 2) signaling pathway [34]. Research further indicates that exenatide reverts the adverse changes to brain derived neurotrophic factor (BDNF) signaling and to the neuroinflammatory status of 3xTg-AD (APPSwe, tauP301L)1Lfa/J) mice receiving a HFD, without affecting systemic metabolism or promoting changes in cognitive performance [35]. Furthermore, HFD feeding decreases cerebral and hepatic low density lipoprotein receptor-related protein 1 gene (LRP-1) expression and elevates cerebral amyloid-β (Aβ) burden without affecting cerebrovascular LRP-1 and IR-β (Insulin Receptor) levels [36]. HFD induces microbiota dysbiosis and defects in spermatogenesis, with the potential causes being elevated endotoxin, dysregulation of testicular gene expression, and localized epididymal inflammation [37]. A recent report showed that a gestational HFD attenuates memory decline, synaptic dysfunction, and Aβ and tau neuropathology in the offspring by transcriptionally regulating BACE-1 (Beta- Secretase 1), CDK5 (Cyclin Dependent Kinase 5), and tau gene expression via upregulation of the FOXP2 (Forkhead box protein P2) repressor [26]. All of these results indicate that brain development in the offspring is influenced by the gut microbiota.

Therefore, in this study, we selected the HSHF diet as a means of interfering with the gut microbiota and metabolism of mother mice, while using epigenetics to monitor the gut microbiota, intestines, and the brain condition of the offspring. Using this approach, we aimed to find out how the gut microbiota affects the neural development of the brain. We also hoped to provide more data toward the knowledge of intestinal and brain interactions, and to provide a reference for diet in pregnant women and for research into developmental nutrition.

## RESULTS

### Changes to the gut microbiota during the growth stage

**Figure 1:**
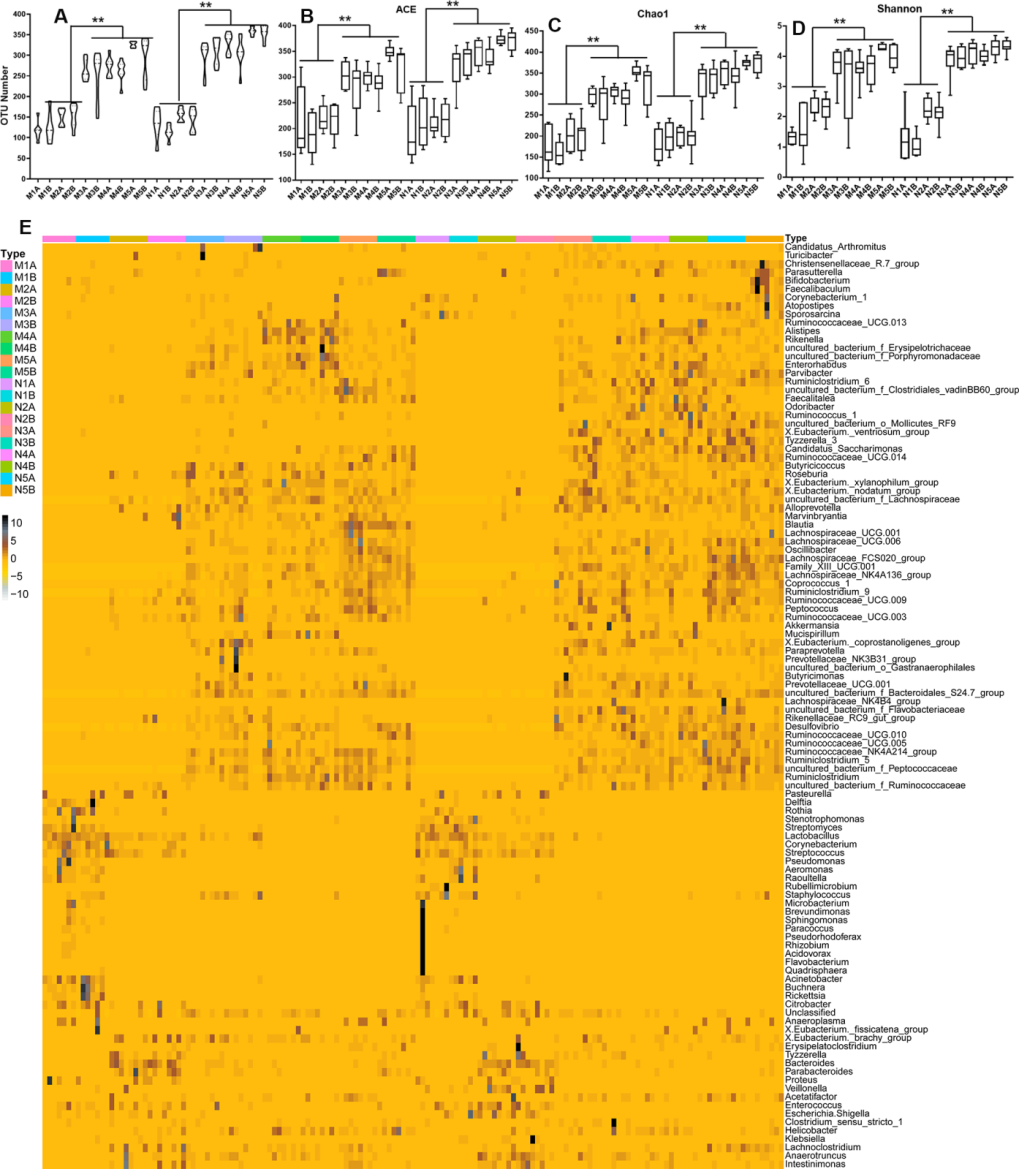
**Influences of maternal high sugar and fat diet on the gut microbiota of offspring during the growth stage.** (**A**) The OTUs number of high sugar and fat diet (HSHF) group were low than in the standard diet; the 16S rRNA analysis showed significant differences for the abundance-based covered estimator (ACE, **B**), Chao 1 (**C**) and Shannon (**D**) indexes (*p* < 0.05); (**E**) the heatmap of genus bacterial of the whole gut microbiota compositions of the offspring birth from the standard diet and HSHF-diet fed mother at different growth phase (7, 14, 21, 28, 56 days). Also see in Supplementary Figures 1–4. The symbol of N1A is the 7-day control male samples, N1B is the 7-day control female samples, and N2A for 14-day, N3A for 21-day, N4A for 28-day, N5A for 56-day male samples, N2B for 14-day, N3B for 21-day, N4B for 28-day, N5B for 56-day female samples; M1A is the 7-day HSHF male samples, M1B is the 7-day HSHF female samples, and M2A for 14-day, M3A for 21-day, M4A for 28-day, M5A for 56-day male samples, M2B for 14-day, M3B for 21-day, M4B for 28-day, M5B for 56-day female samples. Data are presented as the means ± SD of 8 independent experiments. ^*^p <0.05 and ^**^p <0.01 *vs*. the model group by one-way ANOVA, followed by the one-way analysis of variance (ANOVA) at *p* < 0.05.

To study the impact of the maternal diet during pregnancy on the growth, development, health, and disease of the offspring, we compared the microbiota *in fimo* at different growth phases, or from mothers fed on different diets during pregnancy, at least eight samples for each subgroup. The alpha diversity (Figure 1 and Supplementary Figures 1A–1F, 2A, 2C, 3) and the beta diversity (Supplementary Figures 2B, 2D, 3A–3G) could be distinguished between each of the standard diet, high sugar and high fat diet (HSHF), and dietary supplement groups. We first analyzed the constitutive changes between the standard diet groups: the number of operational taxonomic units (OTU) increased from low (35) to high (253) as the offspring grew up (Figure 1A and Supplementary Figures 1, 4A–4G), while the quantity variance between individuals decreased (Supplementary Figure 4A–4G). This indicated that the composition of the gut microbiota became more stable from birth to adulthood, the abundance of bacteria increased, and individual differences gradually decreased. Principal component analysis (PCA) showed that the 7- (N1) and 14- (N2) day samples could be successfully distinguished from the 21- (N3), 28- (N4), and 56- (N5) day samples; after 21 days of age, the differences became smaller (Supplementary Figure 3). The HSHF groups from the different growth phases (at 7 (M1), 14 (M2), 21 (M3), 28 (M4), and 56 (M5) days of age) showed the same variation tendency (Supplementary Figures 2A–2D, 4A–4G).

The heatmap of bacterial genera in Figure 1E shows the whole gut microbiota compositions of the offspring from mothers fed with the standard diet (Supplementary Figure 4A) and the HSHF diet (Supplementary Figure 4B) were similar. The variation tendencies were similar in these two groups, with the relative abundance of the following genera highest at 7 days after birth, before gradually declining as the offspring grew up: *Pasteurella*; *Delftia*; *Stenotrophomonas*; *Streptomyces*; *Lactobacillus*; *Corynebacterium*; *Streptococcus*; *Pseu-domonas*; *Aeromonas*; *Raoultella*; *Rubellimicrobium*; *Staphylococcus*; *Rothia*; *Acinetobacter*; *Buchnera*; *Rickettsia*; and *Citrobacter*. Subsequently bacteria from the following genera grew to become the dominant populations: *Atopostipes*; *Sporosarcina*; *Ruminococc-aceae UCG-013*; *Alistipes*; *Rikenella*; *Erysipelo-trichaceae*; *Porphyromonadaceae*; *Enterorhabdus*; *Parvibacter*; *Ruminiclostridium 6*; *Clostridiales vadin BB60 group*; *Faecalitalea*; *Odoribacter*; *Ruminococcus 1*; *Mollicutes RF9*; [*Eubacterium*] *ventriosum group*; *Tyzzerella 3*; *Candidatus Saccharimonas*; *Ruminoc-occaceae UCG-014*; *Butyricicoccus*; *Roseburia*; [*Eubacterium*] *xylanophilum group*; [*Eubacterium*] *nodatum group*; *Lachnospiraceae*; *Alloprevotella*; *Marvinbryantia*; *Blautia*; *Lachnospiraceae UCG-001*; *Lachnospiraceae UCG-006*; *Oscillibacter*; *Lachno-spiraceae FCS020 group*; *Family XIII UCG-001*; *Lachnospiraceae NK4A136 group*; *Coprococcus 1*; *Ruminiclostridium 9*; *Ruminococcaceae UCG-009*; *Peptococcus; Ruminococcaceae UCG-003*; *Akkerma-nsia; Mucispirillum*; [*Eubacterium*] *coprostanoligenes group*; *Paraprevotella*; *Prevotellaceae NK3B31 group*; *Gastranaerophilales*; *Butyricimonas*; *Prevotellaceae UCG-001*; *Bacteroidales S24-7 group*; *Lachnospi-raceae NK4B4 group*; *Flavobacteriaceae*; *Rikenell-aceae RC9 gut group*; *Ruminococcaceae UCG-010*; *Ruminococcaceae UCG-005; Peptococcaceae*; *Rumino-coccaceae NK4A214 group*; *Ruminiclostridium 5*; *Ruminiclostridium*; *Desulfovibrio*; and *Ruminococc-aceae*.

Further details of the different growth phases given in Supplementary Figure 4A–4G show that the relative abundances and compositions of the different bacterial genera were changed, along with the variances of stages T1 and T2 (7 and 14 days of age) compared with the other stages (21, 28, and 56 days of age). The variances in the offspring born from the HSHF diet were enlarged, indicating that feeding the mother with the HSHF diet during pregnancy influenced the hereditary stability of gut microbiota. As such, individual outliers are more common in the HSHF-diet groups than in the standard-diet groups, especially at the T1 (Supplementary Figure 4C), T2 (Supplementary Figure 4D), and T3 (Supplementary Figure 4E) stages. The heatmap appears a bit chaotic and random at the T4 stage (Supplementary Figure 4F), indicating that the gut microbiota changed dramatically and then gradually stabilized at 1 month of age. This randomness might be induced by weaning or by other factors, but this requires further investigation. After this, the HSHF and standard diet groups were significantly different at the T5 stage, with the following genera increased in the male, standard-diet mice (Supplementary Figure 4G): [*Eubacterium*] *ventriosum group*; *Tyzzerella 3*; *Veillonella*; *Family XIII UCG 001*; *Ruminococcaceae UCG 005*; *Rikenellaceae RC9 gut group*; *Ruminococcaceae UCG 010*; *Lachnospiraceae FCS020 group*; *Ruminococcus 1*; [*Eubacterium*] *coprostanoligenes group*; *Parvibacter*; *Mollicutes RF9*; *Desulfovibrio*; *Lachnospiraceae NK4A136 group; Odoribacter*; *Flavobacteriaceae*; *Lachnospiraceae NK4B4 group*; *Candidatus Saccharimonas*; *Roseburia*; [*Eubacterium*] *brachy group; Ruminococcaceae UCG 014*; *Mucispirillum*; [*Eubacterium*] *nodatum group*; and *Faecalitale*. In contrast, the following genera were increased in the female, standard-diet mice: *Proteus*; *Faecalibaculum*; *Bifidobacterium*; *Christensenellaceae R-7 group*; *Lactobacillus*; *Enterorhabdus*; *Bacteroidales S24-7 group*; *Escherichia/Shigella*; *Alloprevotella*; and *Clostridium sensu stricto 1*. However, the intestinal microbial diversities of the HSHF-diet groups were reduced (Figure 1; *p* < 0.05).

### Changes in autophagy and neurodevelopment during the growth stage

**Figure 2:**
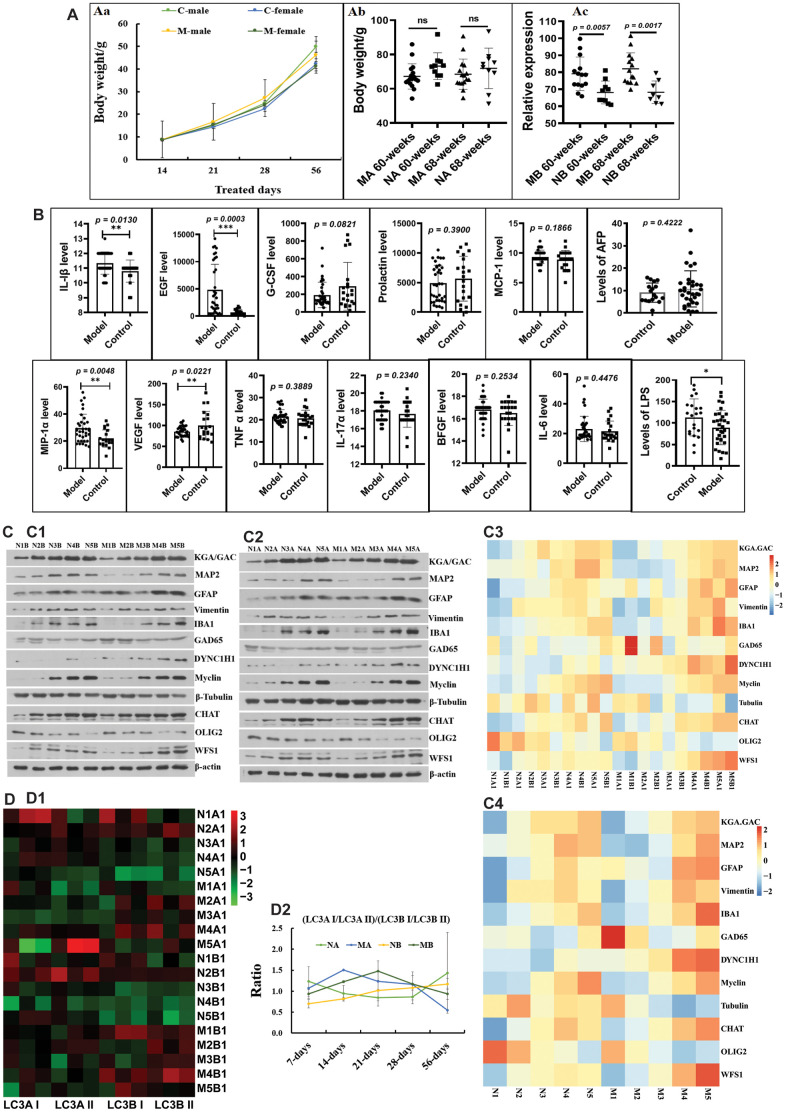
**Influences of maternal high sugar and fat diet on autophagy and neurodevelopment of offspring during the growth stage.** (**A**) Changes on the body weight; (**B**) Changes on inflammatory factors in serum; (**C**) the marked proteins expression of KGA/GAD, MAP2, GFAP, Vimentin, IBA1, GAD65, DYNC1H1, Myclin, Tubulin, CHAT, OLIG2 and WFS1 in brain tissues, (**D**) and the expression LC3A I/LC3A II/ LC3B I/LC3B II protein in brain tissues. The symbol of NA is the 16-month control male samples, NB is the 16-month control female samples; MA is the 16-month HSHF male samples, MB is the 16-month HSHF female samples. Data are presented as the means ± SD of more than 8 independent experiments, and more than 3 independent experiments in Western bolting. ^*^p <0.05 and ^**^p <0.01 *vs*. the model group by one-way ANOVA, followed by the one-way analysis of variance (ANOVA) at *p* < 0.05.

Before 56 days of age, the body weights of the HSHF-diet groups were higher than those of the standard-diet groups (Figure 2Aa). While there were no obvious differences in body weight in adult males (Figure 2Ab), there were significant differences in the body weights of adult females (Figure 2Ac). This indicates that body weight in females is more related to the maternal diet. As they grew older, only a few inflammatory factors, including interleukin 1β (IL-1β), epidermal growth factor (EGF), vascular endothelial growth factor (VEGF), MIP-1α, and lipopolysaccharide (LPS) were changed in the serum (Figure 2B; *p* < 0.05). However, we found that a high serum alpha-fetoprotein (AFP) level was most common in overweight individuals from the HSHF-diet groups (Figure 2B). In addition, we found that some of these individuals had spontaneous tumors, including liver cancer, colorectal cancer, subcutaneous sarcoma, and splenomegaly (data not shown). This suggests that the maternal HSHF diet substantially increases the risk of carcinogenesis and increases body weight in females.

We monitored the development of neurons using the following marker proteins: KGA/GAD; MAP2; GFAP; vimentin; IBA1; GAD65; DYNC1H1; myclin; tubulin; CHAT; OLIG2; and WFS1. As shown in Figure 2C (Figures 2C1–C4), the HSHF diet markedly influences some types of neurons, alongside vimentin and GFAP in astrocytes, and IBA1 in activated microglia (Supplementary Figure 5A). The same trend was observed for CHAT in cholinergic neuron, DYNC1H1 in axons, and WFS1 in the hippocampal CA1 subfield (Figure 2C; *p* < 0.05). In addition, there were some opposing trends, such as for OLIG2 in oligodendrocytes, and tubulin in neurons (Figure 2C; *p* < 0.05). We also monitored the levels of autophagy, as shown in Figure 2D. We found that the ratio of the relative expression levels of LC3A I/LC3A II/ LC3B I/LC3B II protein in the HSHF-diet groups were higher than those in the standard diet groups before 28 days of age, after which they decreased (Figure 2D2). Taken together, these results show that the maternal diet influenced brain development in the offspring.

H&E staining showed the pathological structure of the small intestine (Supplementary Figure 5B), with the pathomorphological changes being different at different growth phases (7, 14, 21, 28, and 56 days of age). This indicates that the gut microbiota or diet can affect the maturation of the intestinal tissue, but the mechanisms underlying this effect need much more in-depth research.

### Long-term potentiation in hippocampal slices

**Figure 3:**
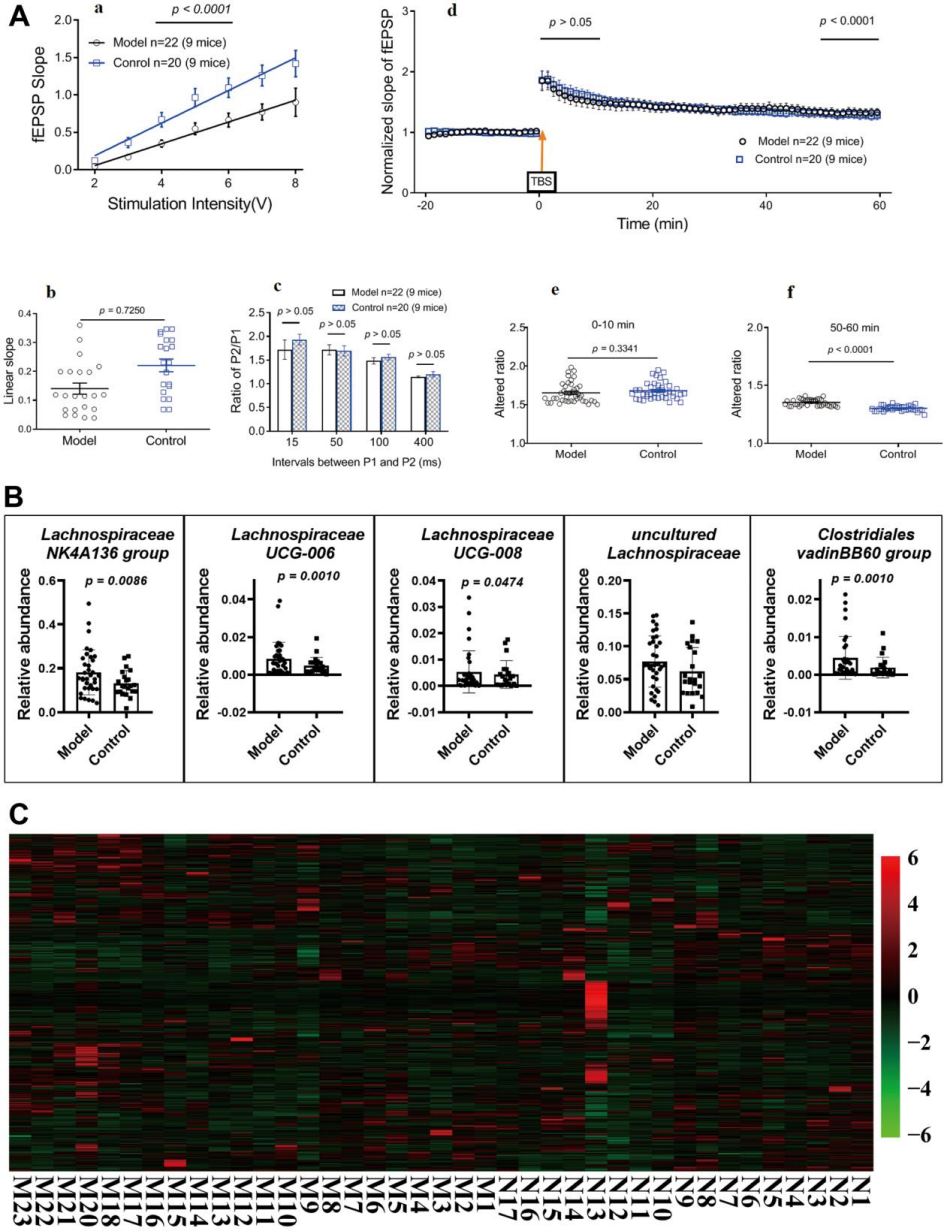
**Influences of maternal high sugar and fat diet on LTP, gut microbiota and metabolome of older mice.** (**A**) LTP in hippocampal slices, 16 months later, subsets of mice were randomly selected to perform standard field potential recordings. (**B**) Changes on the gut microbiota; (**C**) Changes on the metabolome *in fimo*. The pathological structure of brain and small intestine (Supplementary Figure 5). Data are presented as the means ± SD of more than 6 independent experiments. Significant differences between two groups of LTP were evaluated by two-tailed unpaired Student’s t-tests or two-tailed Welch’s t-test. Significant differences between treatments were analyzed by one-way analysis of variance (ANOVA) at *p* < 0.05.

After ten months, subsets of mice were randomly selected to perform standard field potential recordings. Repetitive stimulation (0.33 Hz) of Schaffer collaterals evoked fEPSPs in the hippocampal CA1 region (Figure 3Aa; *p* < 0.05). The input–output relationship curves (Figure 3Ab; *p* > 0.05) and linear slopes (Figure 3Ac; *p* > 0.05) revealed that compared to control mice (standard-diet mice, control; HSHF-diet mice, model), the hippocampal CA1 neurons of model mice showed no differences. Synaptic short-term plasticity was measured using a PPF protocol in hippocampal slices. Statistical analyses of data from all slices demonstrated an inhibition of the ratio of P2/P1 at all tested P1 and P2 intervals in model mice compared to control mice (Figure 3Ad; *p* > 0.05), suggesting HSHF-diet no impaired hippocampal synaptic short-term plasticity. Finally, hippocampal CA1 synaptic LTP were compared between model and control mice. Plotting of the recording time to normalized fEPSP slopes (baseline set as 1) from pooled data showed impaired LTP induction (after theta-burst stimulation at 0–10 min) and maintenance (after theta-burst stimulation at 50–60 min) in all treated groups (Figure 3Ae- *p* > 0.05, unpaired *t* test). The mean LTP induction results are shown in Figures 3Ad and f (*p* < 0.05, unpaired *t* test). This suggests that consuming a HFHS diet before and during pregnancy could improve LTP in the offspring and suggests that the gut microbiota may play an important role.

### Changes in the microbiota and metabolomes of older mice

Analysis with 16S rRNA on some of the mice who were fed until 16 months of age showed that there were no obvious differences in the gut microbiota in the HSHF-diet and standard-diet groups. Only a little of bacteria were changed, as the abundances of *Lachnospiraceae*, *Clostridia*, *Clostridiales*, and *Rikenellaceae* were different, with the following genera more abundant in the HSHF diet groups than in the standard diet groups (Figure 3B; *p* < 0.05): *Lachnospiraceae NK4A136 group*; *Lachnospiraceae UCG-006*; *Lachnospiraceae UCG-008*; and *Clostridiales vadinBB60 group*.

Metabolic analysis of colonic contents from the HSHF-diet mice showed 860 different metabolites via LC/MS (Figure 3C). Pathway analysis of the different metabolites using MetaboAnalyst 4.0 (see metabolome in Supplementary Figure 6A) showed significant differences in the following pathways: Pyrimidine metabolism (*p* = 3.96E-05); Cysteine and methionine metabolism (*p* = 4.14E-05); Sphingolipid metabolism (*p* = 4.14E-05); Aminoacyl-tRNA biosynthesis (*p* = 5.01E-05); Arginine biosynthesis (*p* = 6.32E-05); Purine metabolism (*p* = 6.32E-05); Glycine, serine and threonine metabolism (*p* = 6.72E-05); and Glutathione metabolism (*p* = 9.17E-05).

### Cholinergic and GABAergic neuron counts were increased by upregulation of LHX8 in the HSHF-diet offspring as they grew older

**Figure 4:**
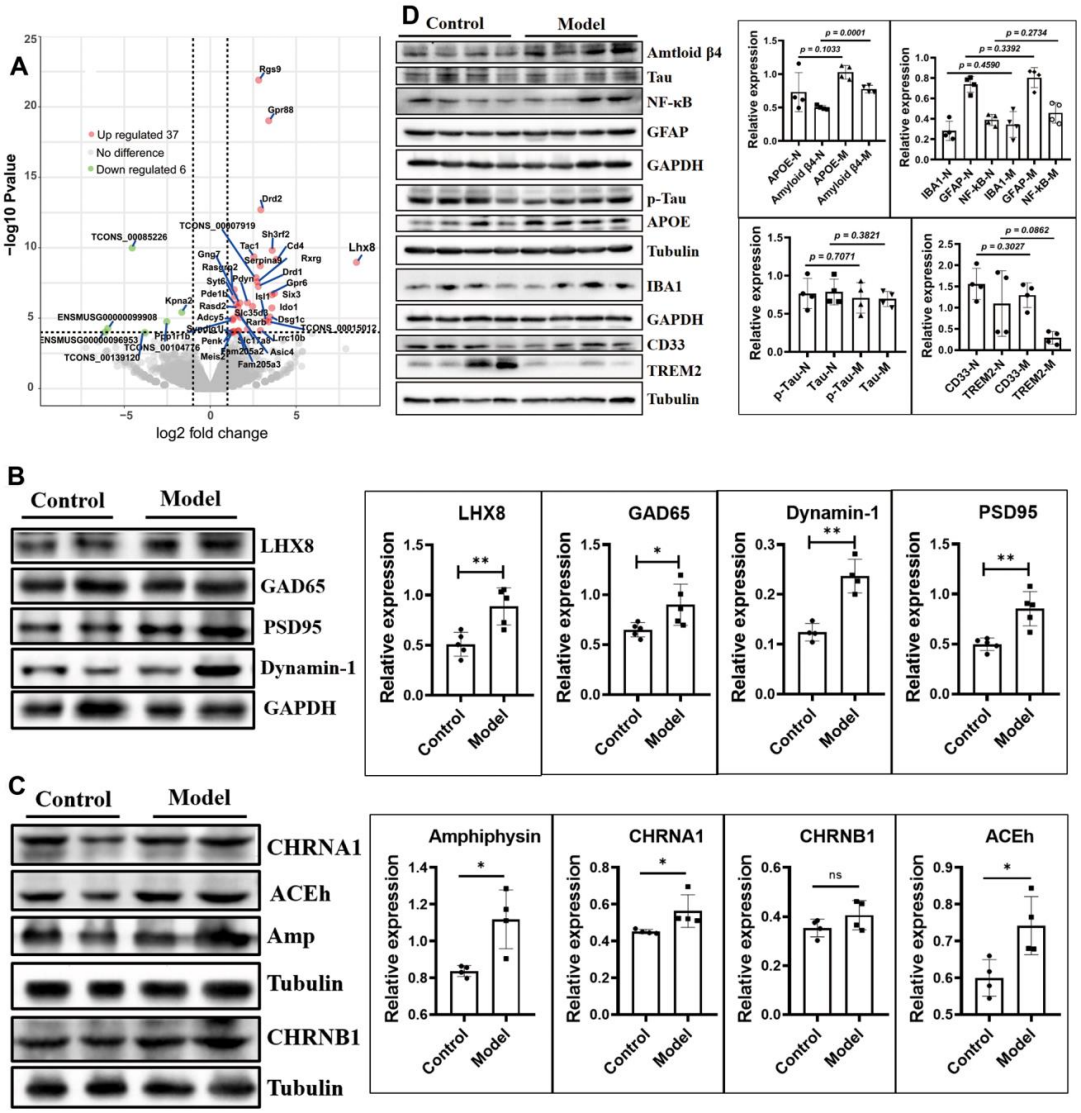
**The cholinergic and GABAergic neurons were increased by up-regulated the LHX8 in the HSHF diet offspring when they getting older.** (**A**) The mRNA sequence of whole brain tissues; (**B**, **C**) the cholinergic and GABAergic cells marked protein of ACEh, Amp, CHRNA1, CHRNB1 and GAD65; the synaptic functional markers of Dynamin 1 and PSD95, and the AD biomarkers of Tau, p-Tau, APOE, CD33 and TREM2 (**D**). The KEGG analysis results see in the Supplementary Figure 6. Data are presented as the means ± SD of more than 3 independent experiments in Western bolting. ^*^p <0.05 and ^**^p <0.01 *vs*. the model group by one-way ANOVA, followed by one-way analysis of variance (ANOVA) at *p* < 0.05.

The mRNA sequence of whole brain tissues showed that 6 mRNAs were downregulated (*TCONS_00085226*, *ENSMUSG00000096953*, *ENSMUSG00000099908, TCONS_00104776*, and *TCONS_00139120*, *Kpna2*), and 37 mRNAs were upregulated, including *Lhx8*, predicted pseudogene 10184 (*Gm10184*), *Cd4*, adenosine A2a receptor (*Adora2a*), synapse differentiation-inducing 1-like (*Syndig1l*), and synaptotagmin VI (*Syt6*) (Figure 4A and Table 1; *p* < 0.05).

**Table 1:** The different expressed mRNA in the offspring of whose mother fed HSHF diet.

**Symbol**	**N_fpkm**	**M_fpkm**	**log2(FC)**	***P*-value**	**FDR**	**Significant**
Rxrg	1.3000	8.5167	2.7308	2.61E-08	3.82E-05	up
Rarb	2.3933	6.8767	1.5945	5.05E-06	3.70E-03	up
Kpna2	14.5300	4.6467	-1.6530	3.77E-06	2.88E-03	down
Slc17a8	0.2867	0.9700	2.1646	6.12E-05	3.33E-02	up
Adora2a	1.3200	17.6900	3.8129	1.26E-27	1.20E-23	up
Rgs9	6.1533	42.7467	2.8179	1.25E-22	7.94E-19	up
Drd1	1.2067	8.0733	2.7902	5.61E-08	7.63E-05	up
Pde1b	34.1267	92.4600	1.5092	9.75E-07	8.85E-04	up
Adcy5	20.4867	48.9767	1.3243	1.07E-05	7.01E-03	up
Cd4	0.2133	3.3833	3.8388	6.68E-10	1.41E-06	up
Meis2	17.3900	41.5400	1.3304	1.01E-04	4.55E-02	up
Pdyn	2.6300	11.3500	2.1636	7.88E-07	7.90E-04	up
Syt6	5.4200	13.7333	1.4156	2.84E-07	3.00E-04	up
Ido1	0.0867	1.0033	3.5852	1.86E-06	1.48E-03	up
Drd2	2.1800	15.8833	2.9403	1.97E-13	7.50E-10	up
Rasgrp2	35.8433	70.9600	1.3913	9.38E-08	1.19E-04	up
Asic4	2.7333	8.6967	1.7342	8.60E-06	5.85E-03	up
Rasd2	14.8867	43.3400	1.6131	1.67E-06	1.38E-03	up
Dsg1c	0.0200	0.2133	3.4806	8.28E-06	5.84E-03	up
Six3	0.2800	3.4967	3.7379	1.73E-07	2.06E-04	up
Isl1	0.2533	2.5767	3.2344	1.49E-05	9.13E-03	up
Penk	97.9967	226.9467	1.2404	1.00E-04	4.55E-02	up
Gpr6	0.5867	6.8467	3.5876	2.19E-07	2.45E-04	up
Gng7	21.5200	71.1867	1.7225	8.33E-07	7.93E-04	up
Slc35d3	0.6433	3.3900	2.4592	1.45E-06	1.25E-03	up
Sh3rf2	0.1867	1.8233	3.6057	1.53E-10	4.15E-07	up
Serpina9	0.7500	5.3200	2.9103	1.91E-09	3.30E-06	up
Ppp1r1b	71.8133	167.5233	1.3000	1.34E-05	8.53E-03	up
Tac1	9.1000	51.9433	2.5360	4.38E-10	1.04E-06	up
Gm10184	0.1733	10.7800	5.9345	9.93E-29	1.89E-24	up
Gpr88	4.2200	53.4033	3.4044	9.59E-20	4.57E-16	up
Syndig1l	6.5100	15.8800	1.3751	8.42E-05	4.11E-02	up
Lrrc10b	2.2600	16.3133	2.9145	7.49E-05	3.96E-02	up
Fam205a3	1.6233	4.7567	1.6366	7.94E-05	3.98E-02	up
Fam205a2	1.6233	4.7567	1.6366	7.94E-05	3.98E-02	up
Lhx8	0.0010	1.7533	8.5055	1.08E-09	2.07E-06	up
TCONS_00007919	0.4000	2.4333	2.6665	1.31E-08	2.08E-05	up
TCONS_00015012	0.0633	0.7033	3.4096	1.82E-05	1.05E-02	up
TCONS_00085226	1.3800	0.0533	-4.5407	1.03E-10	3.28E-07	down
TCONS_00104776	49.1267	8.1100	-2.5287	1.75E-05	1.04E-02	down
TCONS_00139120	0.7967	0.0533	-3.8060	1.03E-04	4.55E-02	down

*L3/Lhx8* is involved in the determination of cholinergic or GABAergic cell fate [27]. The mRNA sequence results showed that expression of *Lhx8* (Figure 4A and Table 1; *p* < 0.05) and the LHX8 protein (Figure 4B, *p* < 0.05) were upregulated. Cholinergic and GABAergic cells regulated by this protein showed upregulated levels of the marker proteins ACEh, Amp, CHRNA1, CHRNB1, and GAD65 (Figure 4B, 4C, *p* < 0.05).

Expression of the synaptic functional markers dynamin 1 and PSD95 differed (Figure 4B; *p* < 0.05), while the AD biomarkers Tau, p-Tau, APOE, CD33, and TREM2 showed no obvious differences in expression (Figure 4D; *p* > 0.05), except for Aβ_42_ (Figure 4B, *p* = 0.0001). Furthermore, levels of IBA1, GFAP, and NF-κB did not differ (Figure 4D; *p* > 0.05), indicating that the maternal HSHF-diet had no obvious harmful effects on the offspring in terms of AD.

The mRNAs of *Rgs9*, *Gpr88*, *Drd2*, *Sh3rf2*, *Tac1*, *Serpina 9*, *Rxrg*, *Drd1*, *Rasgrp2*, *Six3*, *Gpr6*, *Pdyn*, *Gng7*, and *Pde1b* were all upregulated (Figure 4A and Table 1; *p* < 0.05); all of these have been reported to be more or less related to nervous system diseases. Kyoto Encyclopedia of Genes and Genomes (KEGG) pathway analysis showed that significantly altered pathways, including: Dopaminergic synapse; cAMP signaling pathway; Gap junction; Rap1 signaling pathway; Retrograde endocannabinoid signaling; Glutamatergic synapse; Neuroactive ligand-receptor interaction; and Calcium signaling pathway (Supplementary Figure 6B, 6C).

### Special maternal diet changes the gut microbiome trace

The use of specific dietary supplements (*Hericium erinaceus* and *Ganoderma lucidum*) during pregnancy changed the inheritance of microorganisms (Supplementary Figures 2A, 2B). In addition, exposure to different microorganisms in early life from the outdoor soil (T-) and from SAMP8 mice (S-) also changed the inherent abundances of different species (Supplementary Figures 2C, 2D).

**Figure 5:**
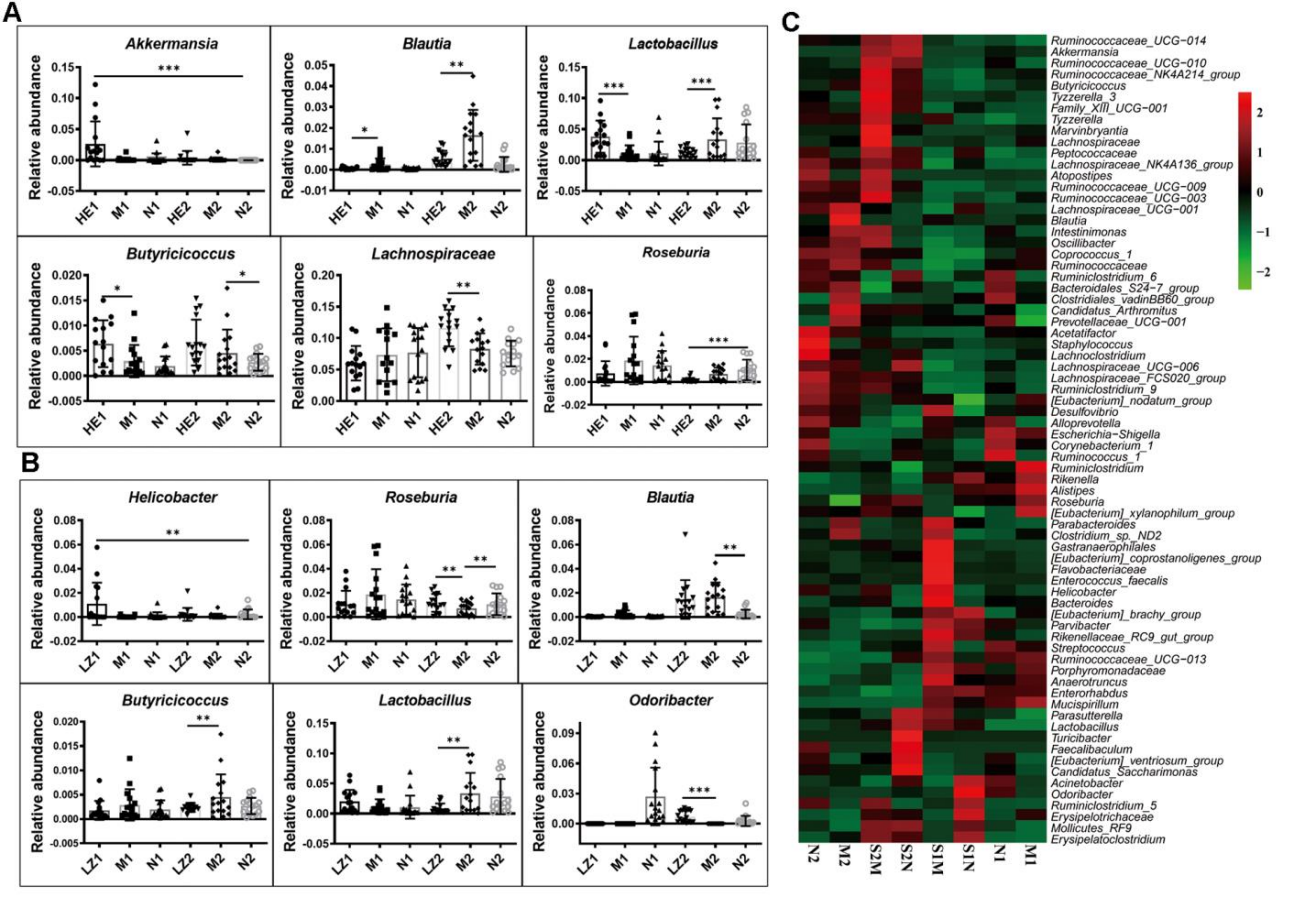
**Special maternal diet changes the gut microbiome trace.** (**A**) The dietary supplement of *Hericium erinaceus* group (**A**); the dietary supplement of *Ganoderma lucidum* (**B**); the heatmap of genus bacterial influenced by the postpartum environmental exposure (**C**). The symbol of N1 is the 28-day control samples, N2 is the 56-day control samples; M1 is the 28-day HSHF samples, M2 is the 56-day HSHF samples; *HE1* is for *Hericium erinaceus* treated 28-day samples, *HE2* is for *Hericium erinaceus* treated 56-day samples, *LZ1* is for *Ganoderma lucidum* treated 28-day samples, *LZ2* is for *Ganoderma lucidum* treated 56-day samples. Data are presented as the means ± SD of more than 8 independent experiments. ^*^p <0.05 and ^**^p <0.01 *vs*. the model group by one-way ANOVA, followed by the one-way analysis of variance (ANOVA) at *p* < 0.05. Also see in the Supplementary Figures 7, 8

The heatmap of bacterial genera in Supplementary Figure 7 shows that the whole gut microbiota compositions of the offspring born from mothers fed on a specific dietary supplement during pregnancy were similar, and their variation tendencies were also the same. However, the relative abundances of some bacterial genera were very different: those of *Akkermansia*, *Blautia*, *Butyricicoccus*, *Lactobacillus*, *Lachnospiraceae*, and *Roseburia* were changed at 28 days and/or 56 days in the *Hericium erinaceus* dietary supplement group (Figure 5A); and *Helicobacter*, *Odoribacter*, *Blautia*, *Butyricicoccus*, *Lactobacillus*, and *Roseburia* were changed in the *Ganoderma lucidum* dietary supplement group (Figure 5B). This indicates that specific dietary supplement with *Hericium erinaceus* or *Ganoderma lucidum* changes the gut microbiota composition induced by the HSHF diet.

The heatmap of bacterial genera in Supplementary Figure 8 shows that the gut microbiota compositions are also influenced by postpartum environmental exposure, with the relative abundances of the following genera significantly changed after co-housing during pregnancy (Figure 5C): *Parabacteroides*; *Clostridium sp. ND2*; *Gastranaerophilales; Enterococcus faecalis*; *Helicobacter*; [*Eubacterium*] *coprostanoligenes group*; *Flavobacteriaceae*; *Bacteroides*; *Parasutterella*; *Lactobacillus*; *Turicibacter*; *Faecalibaculum*; [*Eubac-terium*] *ventriosum group*; *Candidatus Saccharimonas*; *Acinetobacter*; *Odoribacter*; *Ruminiclostridium 5*; *Mollicutes RF9*; *Erysipelotrichaceae*; and *Erysipelato-clostridium* (more details are shown in Supplementary Figure 8). Influences of gut microbiota on brain function were shown in our previous paper [38].

All of these results indicate that the diet during pregnancy is very important; however, the majority of the functions of the bacteria remain unknown.

### Choline plays an important role in the cholinergic system

**Figure 6:**
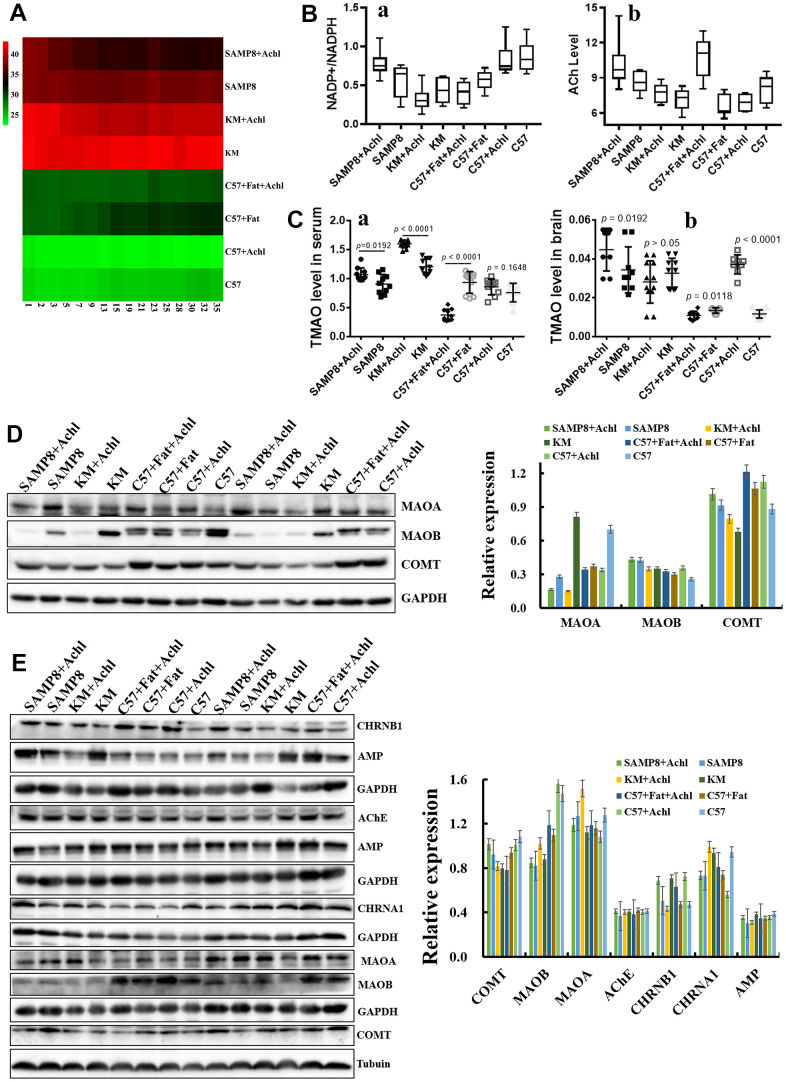
**Gut microbiota plays an important role in the bioconversion of choline into TMAO and Ach.** (**A**) The body weight changes after administration of choline; (**B**) Levels of acetylcholine and NADPH/NADP+ in the brain samples after administration of choline; (**C**) TMAO levels in serum and brain tissues after administration of choline mice; (**D**) Expression of the proteins MAOA, MAOB and COMT in liver; (**E**) Expression of the proteins MAOA, MAOB and COMT in brain tissues, and expression of the proteins AChE, AMP, CHRNA1 and CHRNB1 in the brain tissues. Data are presented as the means ± SD of more than 8 independent experiments, and more than 3 independent experiments in Western bolting. ^**^p <0.05 and ^*^p <0.01 *vs*. the control group by one-way ANOVA test.

The cholinergic hypothesis plays an important role in AD therapy. To investigate the role of the gut microbiota on the conversion rate of choline, 50 mg/kg/d choline was fed to mice with different gut microbiota, including SAMP8 mice, KM mice, C57 mice, and HSHF-diet mice (at 5 weeks of age). As shown in Figure 6, body weight was slightly lowered after treatment with choline (Figure 6A), and levels of acetylcholine (Figure 6B; *p* < 0.05) and NADPH/NADP^+^ (Figure 6B; *p* < 0.05) were also changed. In addition, TMAO levels in serum (Figure 6Ca) and in brain tissue (Figure 6Cb) were changed, especially in mice pre-fed the HSHF diet (*p* < 0.05). Expression levels of the proteins MAOA, MAOB, and COMT in liver (Figure 6D) and in brain (Figure 6E; *p* < 0.05) tissues were changed; in particular, choline significantly increased the levels of these proteins in the liver of SAMP8 mice (Figure 6D; *p* < 0.05). Expression of the proteins AChE, AMP, CHRNA1, and CHRNB1 in the brain were also affected (Figure 6E). Together, these observations indicate that the gut microbiota influence the conversion rate of choline to TMAO, and that the TMAO level influences the cholinergic system. Furthermore, the effect of a decrease in body weight was confirmed in this study.

## DISCUSSION

In this study, we showed that the HSHF diet significantly affects the gut microbiota structure of offspring, especially during the early life stage. In addition, in the offspring the HSHF diet influences various types of neuron including cholinergic and GABAergic neurons, the level of autophagy in the brain, and the level of inflammation in the intestinal tract. As the offspring became older (16 months of age), we found that some genes of benefit to nervous system disease were activated, such as *Lhx8*, *GPR88*, *RGS9*, *CD4*, *DRD2*, *RXRG*, and *Syt6*, following the increase in the number of cholinergic and GABAergic neurons. Although the levels of inflammation in the nervous and peripheral systems showed no obvious difference, the number of high AFP level individuals was far higher in the HSHF-diet group than in the standard-diet group. This suggests that more accurate and/or personalized nutrition is needed. Taken together, these results show that the maternal HSHF diet benefits the offspring by reducing the risk of nervous diseases via activation of LHX8, which modulates cholinergic and GABAergic neurons via the gut–brain axis.

Diet is an indispensable factor for the diversity and stability of the gut microbiota, with the nutrients in the food being an important fuel for the gut microbiota [15, 22, 37]. As such, we can adjust the intestine by adjusting the diet and balancing the nutrition affecting microbiota structure [8]. Pregnant women who eat different diets during pregnancy have different gut microbiota and nutritional status, which will affect the gut microbiota of future generations [22]. When a pregnant woman consumes a HSHF diet too much, it will affect the microbiota of the offspring and increase the risk of disease in the future generation. Chu et al. found that in pregnant women, a high-fat diet can influence the offspring and lead to the depletion of *Bacteroides* and the enrichment of *Enterococcus* in the meconium; meanwhile, there is a tendency to reduce bacterial genera at 6 weeks of age [5]. Dietary fiber in food is very beneficial to our health, and increasing the intake of dietary fiber and the concentration of small organic acids (SCAFs) in the body can enrich functional bacteria (comprising 15 strains of acetic acid- and butyric acid-producing bacteria) and improve type 2 diabetes [39]. In contrast, a lack of fiber can lead to a reduction of gut microbiota, which affects the transmission of the gut microbiota from the mother to future generations. Once these effects have been passed on to future generations, simply restoring the dietary fiber intake is not enough to restore the complex gut microbiota [40]. In our study, we found the same changes, with the HSHF diet significantly affecting the gut microbiota structure of the offspring, especially during the early life stage (Figure 1). The autophagy and neurodevelopment of the offspring during the early growth stage were also changed (Figure 2). These changes indicate that diet can influence bacterial genetics and the development of the brain; however, more data and in-depth evaluations are still needed.

Diet during pregnancy affects neurodevelopment in the offspring [4, 5, 41]. The process from pregnancy to the birth of the baby is the first stage of neurodevelopment. Fundamental processes such as cortical and gray matter volumetric growth, neurogenesis, axonal and dendritic growth, synaptogenesis, and myelination begin as early as 20 weeks during gestation [21, 26]. The microbiome of a pregnant woman is closely related to the neurodevelopment of the infant [5, 23, 42]. Current nutrition research needs to pay more attention to diet during pregnancy to intervene in the early life development of infants. The consumption of a large amount of high-fat foods during pregnancy by obese pregnant women will affect the gut microbiota and nervous system of their offspring [5, 21, 23, 26, 41, 42]. A previous study showed that the content of *Lactobacillus* was significantly lower in mice born from high-fat diet mothers than that of normal mice; moreover, the nervous systems of these mice were disordered and developmental disorders (such as autism spectrum disorder), and these behavioral disorders could be reversed by re-adding *Lactobacillus* into their intestines [4]. However, recent studies have shown that high-fat intake in mothers during pregnancy can protect the offspring from the effects of AD in AD model mice, which show better learning and memory, and lower amyloid levels than the offspring with normal diets [26]. Ingesting a high-fat diet leads to the above two very different results; in fact, a correct high-fat diet involves removing unhealthy and excess trans-fatty acids and fat-soluble “garbage” increasing the intake of high-quality unsaturated fatty acids, and improving fat-soluble nutrients, which increases the absorption utilization rate [1]. In addition, supplementation with a choline diet during pregnancy effectively ameliorates the cognitive impairment of APP/PS1 mice and their progeny and improves their memory [43]. In our study, we also found that such a diet can effectively improve the cholinergic system (Figure 6). Furthermore, the supplementation of short-chain fatty acids can regulate microglial homeostasis [21]. A recent study from 2020 found that short-chain fatty acids can determine the differentiation of nerve, intestinal, and pancreatic cells via embryonic GPR41 and GPR43 [22]. In the present study, we found that some genes of benefit to nervous system disease were activated (such as *Lhx8*, *GPR88*, *RGS9*, *CD4*, *DRD2*, *RXRG*, and *Syt6*), following the increase in the number of cholinergic and GABAergic neurons in the HSHF-diet offspring (Figure 4).

Nerve development is regulated by the gut microbiota. Defects in neurodevelopment can lead to deformities and to various sensory, motor, and cognitive impairments, including whole brain deformities and other neurological diseases in humans [22]. Gut microbiota and microbial metabolites in the human body affect the development of nerves through microbial metabolism, immune system regulation, and vagal nerve activation [5, 21, 23, 26, 41, 42]. Microbial metabolites control the activation of microglia and the production of TGF-α and VEGF-β through an aromatic hydrocarbon receptor-mediated mechanism, and regulate the transcriptional program of astrocytes and inflammation of the central nervous system [44]. When mice are born under germfree conditions, microglia in the brain become damaged [21]. The blood–brain barrier ensures the stability of the central nervous system environment. In 2014, Braniste et al. found that the gut microbiota of female mice can affect the development of the blood–brain barrier of embryonic mice [45], with the blood–brain barrier of germfree female offspring “leaking” until adulthood. In addition, common neurological diseases are accompanied by the participation of gut microbiota, such as: AD [24, 25]; Parkinson’s disease [46]; schizophrenia [42]; and autism spectrum disorder [47]. However, aseptic mice or mice in which antibiotics have been used to induce gut microbiota disturbances have impaired cognition and disrupted neurodevelopment [23]. Therefore, the practice of treating gut microbiota and their metabolites will be an effective method of improving nerve development and of treating neurological diseases.

Many studies have reported that autophagy increases the lifespan of model organisms [20, 48]. A previous study uncovered a privileged interaction between the microbiota and mucosal-associated invariant T (MAIT) cells, which sequentially controls both tissue-imprinting and subsequent responses to injury [41]. In this study, we also found that the gut microbiota changed (Figure 1), and that levels of autophagy (Figure 2) and the expression of marker proteins of various types of neurons (Figure 2) were simultaneously changed. Taking all the results together, we conclude that the maternal HSHF diet has benefits for the offspring by reducing the risk of nervous diseases via activation of LHX8, which modulates cholinergic and GABAergic neurons via the gut–brain axis. Furthermore, we conclude that the maternal HSHF diet influences how the microbiome matures in the early life of the offspring, but many of the functions and mechanisms underlying this need further study.

## MATERIALS AND METHODS

### Animal model establishment and treatment

Adult male KM mice (18–22 g, 6 weeks) obtained from the Center of Laboratory Animals of Guangdong Province (SCXK [Yue] 2008-0020, SYXK [Yue] 2008-0085) were pair-housed in plastic cages in a temperature-controlled (25 ± 2° C) colony room under a 12/12-h light/dark cycle. Food and water were available *ad libitum*. All experimental protocols were approved by the Center of Laboratory Animals of the Guangdong Institute of Microbiology. All efforts were made to minimize the number of animals used.

Mice were randomly allocated into two groups: control and model. The mice in the control group were fed with the standard diet, and the mice in the model group were fed a HSHF diet, and water was available freely. Mother mice were fertilized after feeding for 1 month, and continued to be fed on the HSHF diet until birth before returning back to the standard diet. All the offspring follow were fed with the standard diet. The gut microbiota, and intestinal and brain functions of the offspring were dynamically monitored at 7, 14, 28, and 56 days of age, until 16 months of age.

The components of high sugar and fat diet include 20% of sucrose, 15% of fat, 1.2% of cholesterol and 0.2 % of bile acid sodium, 10% of casein and 0.6% of calcium hydrogen phosphate, 0.4% of stone powder, 0.4% of premix, 52.2% of basic feed. Heat ratio: protein 17%, fat 17%, carbohydrate 46%.

### Microbiome analysis

Fresh intestinal content samples were collected from the mouse before it fasted for 12 h, and stored at -80° C. Microbial DNA isolated from the samples, with total masses ranging from 1.2 to 20.0 ng, were stored at -20° C. Microbial 16S rRNA genes were amplified using the forward primer 5′-ACTCCTACGGGAGGCAGCA-3′ and the reverse primer 5′-GGACTACHV GGG TWTCTAAT-3′. Each amplified product was concentrated via solid-phase reversible immobilization and quantified by electrophoresis using an Agilent 2100 Bioanalyzer (Agilent Technologies, Santa Clara, CA, USA). After NanoDrop quantification of the DNA concentration, each sample was diluted to a concentration of 1 × 10^9^ molecules/μL in TE buffer and pooled. Twenty μL of the pooled mixture was sequenced on an Illumina MiSeq sequencing system (Illumina, San Diego, CA, USA) according to the manufacturer’s instructions. Raw pyrosequencing reads obtained from the sequencer were denoised using Titanium PyroNoise software. The resulting pyrosequencing reads were analyzed by common analysis methods, as described previously [38].

### Metabolome analysis

After acquiring serum from the mouse, 80 μL serum was added to 240 μL cold methanol/acetonitrile (2:1, v/v). 10 μL internal tagging standard (L-2-chlorine-phenylalanine, 0.3 mg/mL, dissolved in methanol) was added, the samples were vortexed for 2 min, and then ultrasonic extraction was performed for 5 min. The samples were allowed to stand at -20° C for 20 min, and then centrifuged for 10 min (14,000 RPM, 4° C). 200 μL of the supernatant was loaded into a sample bottle with a lining tube for LC/MS analysis. A Waters UPLC I-class system equipped with a binary solvent delivery manager and sample manager, coupled to a Waters VION IMS Q-TOF Mass Spectrometer equipped with an electrospray interface (Waters Corporation, Milford, MA, USA) was used for LC/MS analysis. An Acquity BEH C18 column (100 mm × 2.1 mm; i.d., 1.7 μm; Waters, Milford, USA) was used for LC separation. Information on the peak picking, alignment, deconvolution, and further processing of raw LC-MS data can be found in previously published protocols [35, 40] (Sah et al. 2017; Sonnerburg et al., 2016).

### Electrophysiological recordings

Standard field potential recordings were performed on the hippocampal cornu ammonis 1 (CA1) region using borosilicate glass micropipettes pulled to a tip diameter of about 1 μm and filled with 2 mol/L NaCl [49]. To record synaptic potentials, a recording electrode was placed at the CA1 apical dendrite region. Stimulus intensity was set based upon input–output relationships and was 50% of the maximal response. For testing paired pulse facilitation (PPF), two stimuli with 50% of the maximal intensity were given at 15-, 50-, 100-, and 400-ms intervals. For recording long-term potentiation (LTP), stable baseline synaptic potentials (50% of the maximal intensity) were recorded for 20 min, and then a theta-burst tetanic stimulation that contained 15 burst trains at 5 Hz was delivered (each train contained five pulses at 100 Hz). Thereafter, baseline intensity-evoked field excitatory postsynaptic potentials (fEPSPs) were recorded for 60 min with 0.33 Hz. A custom bipolar platinum wire electrode (0.08-mm diameter) was placed at the Schaffer collateral pathway, and stimulation was delivered using a Model 2100 A-M Systems Isolated Pulse Stimulator (Carlsborg, WA, USA). All evoked responses were recorded using an Axoclamp-2B amplifier, and data acquisition was controlled with pClamp 10.2 software (Molecular Devices, Sunnyvale, CA, USA).

### Western blot analysis

In order to target the proteins, we used tandem mass tag (TMT) labeling for quantitative proteomic analysis. Global brain tissue was dissected from the offspring of high-fat-diet mice and proteins were extracted with radioimmunoprecipitation assay (RIPA) lysis buffer. Then, total protein samples (from the offspring of the model and control groups) were prepared. The proteins were separated by sodium dodecyl sulfate-polyacrylamide gel electrophoresis and transferred onto polyvinylidene fluoride membranes. After blocking with 5% nonfat dry milk in Tris-buffered saline (20 mM Tris-HCl, 500 mM NaCl, pH 7.4) with 0.2% Tween-20 (Aladdin, T104863), the membranes were probed with antibodies overnight at 4° C, followed by incubation with a horseradish peroxidase-conjugated goat anti-mouse (Servicebio; G2211-1-A) or goat anti-rabbit (Servicebio; G2210-2-A) IgG secondary antibody (1:2,000). Band intensity was quantified using ImageJ software (NIH).

The antibody of KGA/GAD (Proteintech, 12855-1-AP, 1:600), MAP2 (Proteintech, 17490-1-AP, 1:1000), GFAP (Proteintech, 20746-1-AP, 1:2000), vimentin (Proteintech, 10366-1-AP, 1:5000), IBA1 (Proteintech, 10904-1-AP, 1:1000), GAD65 (Proteintech, 20746-1-AP, 1:1000), DYNC1H1 (Proteintech, 12345-1-AP, 1:1000), myelin (Proteintech, 10458-1-AP, 1:3000), tubulin (Abcam, ab7291, 1:10000), CHAT (Proteintech, 207471-1-AP, 1:1000), OLIG2 (Proteintech, 13999-1-AP, 1:400), WFS1 (Proteintech, 11558-1-AP, 1:600), LC3A (CST, 4599S, 1:5000), LC3B (CST, 3868S, 1:5000), ACEh (Proteintech, 17975-1-AP, 1:1000), Amp (Proteintech, 13379-1-1P, 1:2000), CHRNA1 (Proteintech, 10613-1AP, 1:1000), CHRNB1 (Proteintech, 11553-1-AP, 1:1000), Tau (Affinity, AF6141, 1:1000), p-Tau (CST, 11837S, 1:1000), APOE (Absic, 5841, 1:1000), CD33 (Absic, AB32577, 1:1000), TREM2 (Abcam, ab86491, 1:500), LHX8 (Affinity, DF6778, 1:1000), PSD95 (Affinity, AF5283, 1:1000) and NF-κB (Abcam, 16502, 1:1000), MAOA (Proteintech, 10539-1-AP, 1:1000), MAOB (Proteintech, 12602-1-AP, 1:1000), and COMT (Proteintech, 14754-1-AP, 1:500) were measured.

### RNA sequencing

We prepared RNA sequencing libraries using whole brain samples and performed 150-bp paired-end sequencing using an Illumina HiSeq platform (Illumina). RNA sequencing (RNA-seq) libraries were prepared from 2 μg of total RNA using the TruSeq Kit (Illumina), with the modifications. Instead of purifying poly-A RNA using poly-dT primer beads, we removed rRNA using the Ribo-Zero rRNA Removal Kit (Illumina). All other steps were performed according to the manufacturer’s protocols. RNA-seq libraries were analyzed for QC; the average size of inserts was approximately 200 to 300 bp. The sequencing library was then sequenced on the HiSeq platform.

### Histopathology and immunostaining

The brain and intestine were removed and fixed in 4% paraformaldehyde at pH 7.4 for further observation of the pathology. Tissue samples were prepared as paraffin sections after drawing materials, fixation, washing, dehydration, transparency, dipping in wax, and embedding. Immunofluorescent staining of microglia and astrocytes was performed using antibodies to IBA-1, and paraffin-embedded 3-μm sections were stained with hematoxylin-eosin (H&E). Slides were observed using a light microscope and a fluorescence microscope.

### Statistical analysis

All data are described as the mean ± standard deviations (SD) of at least three independent experiments. Significant differences between two groups of LTP were evaluated by two-tailed unpaired Student’s t-tests or two-tailed Welch’s t-test. Significant differences between treatments were analyzed by one-way analysis of variance (ANOVA) at *p* < 0.05 using the Statistical Package for the Social Sciences software (SPSS; Abacus Concepts, Berkeley, CA, USA) and Prism 5 software (GraphPad, San Diego, CA, USA).

### Study limitations

There are several strengths of this study, but there are also limitations. For example, the study lacks a rescue test with single bacteria. As such, we have not identified one-on-one contacts between LHX8 and single bacteria, and therefore could not determine the signaling pathway or linking ligament responsible. Positive verification of this in future could use multi-omics studies of targeted strains in germfree mice to reveal the interaction of LHX8 with the microbiome–gut–brain axis, or use knockout LHX8 mice, to prove the high sugar and fat diet activating *LHX8* to modulate cholinergic and GABAergic neurons via the gut-brain axis.

### Consent for publication

No human data are reported in this manuscript.

### Ethics approval

The animal protocols used in this work were approved by the Institutional Animal Care and Use committee of the Center of Laboratory Animals of the Guangdong Institute of Microbiology.

## Supplementary Material

Supplementary Figures

Supplementary Table 1
