# Correction for: Maternal high sugar and fat diet benefits offspring brain function via targeting on the gut–brain axis

**DOI:** 10.18632/aging.206229

**Published:** 2025-03-31

**Authors:** Dongdong Wang, Haiting Zhang, Miao Zeng, Xiaocui Tang, Xiangxiang Zhu, Yinrui Guo, Longkai Qi, Yizhen Xie, Mei Zhang, Diling Chen

**Affiliations:** 1 State Key Laboratory of Applied Microbiology Southern China, Guangdong Provincial Key Laboratory of Microbial Safety and Health, Guangdong Institute of Microbiology, Guangdong Academy of Sciences, Guangzhou 510070, Guangdong, China; 2 Guangdong Second Provincial General Hospital, Guangzhou 510000, Guangdong, China; 3 Chengdu University of Traditional Chinese Medicine, Chengdu 610075, Sichun, China; 4 Academy of Life Sciences, Jinan University, Guangzhou 510000, Guangdong, China

**Keywords:** maternal diet, pregnancy nutrition, gut-brain axis, cholinergic neurons, GABAergic neurons

**This article has been corrected:** The authors recently found that there was overlap between two H&E images in **Supplementary Figure 5B**. Specifically, the image M4A, which represents the pathological structure of the small intestine in male mice at 28 days, was also unintentionally used for the M3B image, which represents a female sample at 21 days. The authors have replaced the incorrect image with the correct original image from the M3B H&E staining group and stated that this correction does not affect the experimental outcome or conclusions of the study. The authors sincerely apologize for any inconvenience caused.

The corrected version of **Supplementary Figure 5** is provided below.

**Supplementary Figure 5:**
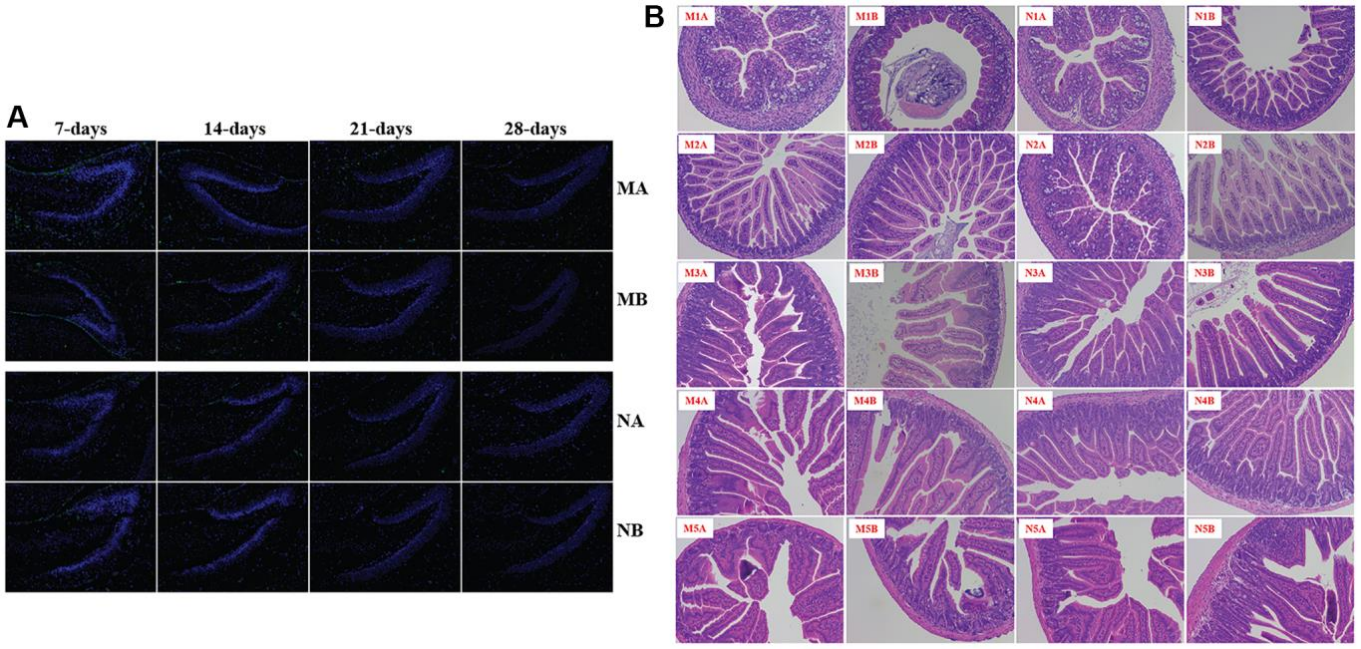
**The pathological structure of brain and small intestine.** IBA1 for microglia (**A**); H&E staining showed the pathological structure of the small intestine (**B**). The symbol of N1A is the 7-day control male samples, N1B is the 7-day control female samples, and N2A for 14-day, N3A for 21-day, N4A for 28-day, N5A for 56-day male samples, N2B for 14-day, N3B for 21-day, N4B for 28-day, N5B for 56-day female samples; M1A is the 7-day HSHF male samples, M1B is the 7-day HSHF female samples, and M2A for 14-day, M3A for 21-day, M4A for 28-day, M5A for 56-day male samples, M2B for 14-day, M3B for 21-day, M4B for 28-day, M5B for 56-day female samples.

