# Supplementary material for: Maternal high sugar and fat diet benefits offspring brain function via targeting on the gut–brain axis
Source: Aging (Albany NY). 2021 Mar 26;13(7):10240–74. doi: 10.18632/aging.202787 (PMC8064210; doi:10.18632/aging.202787)
Supplement: Supplementary Figures [file aging-13-202787-s001.pdf]

SUPPLEMENTARY FIGURES

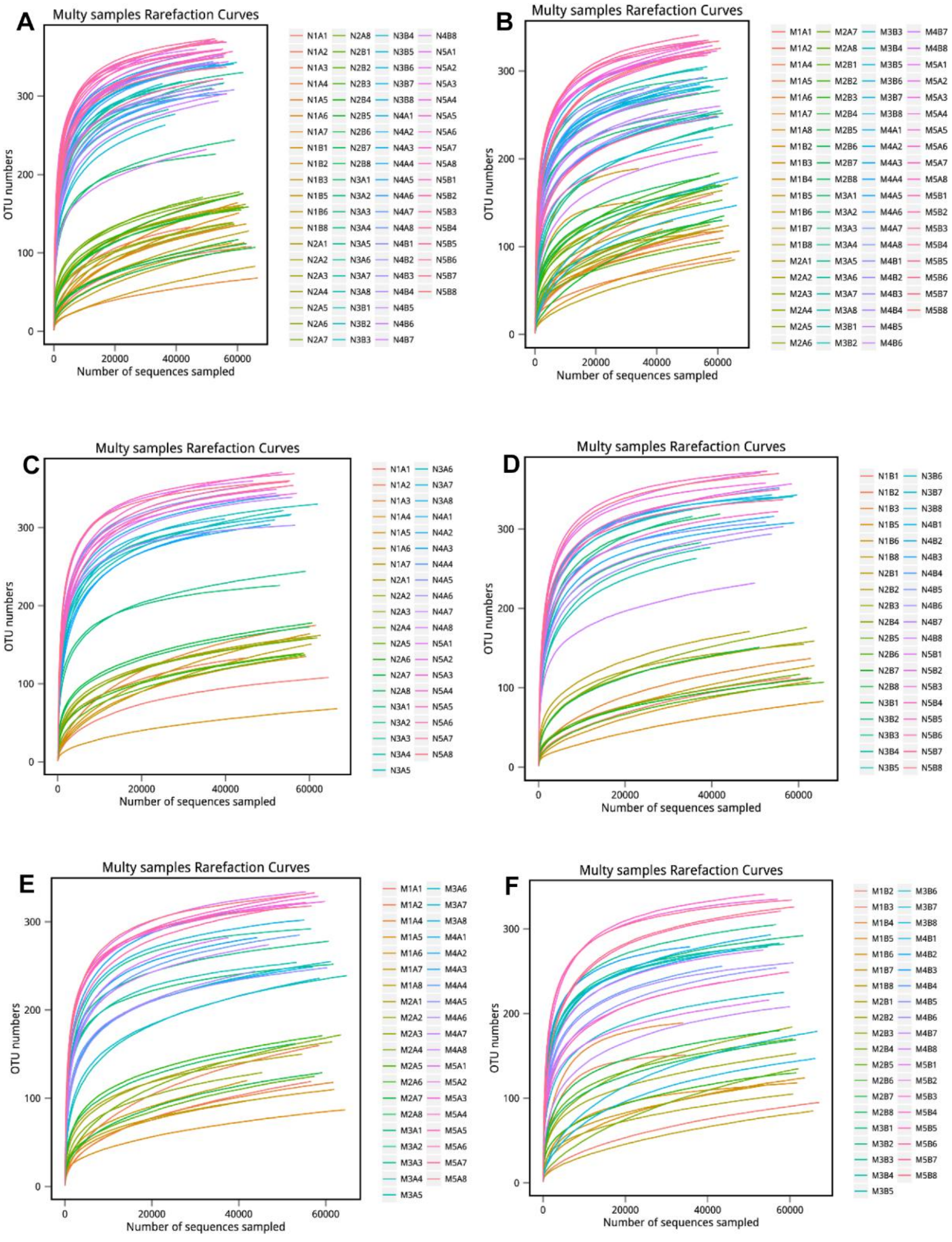

Supplementary Figure 1. Influences of maternal high sugar and fat diet on the alpha diversity of gut microbiota of offspring. (**A**) All the control samples; (**B**) All the HSHF fed samples; (**C**) All the control male samples; (**D**) All the control female samples; (**E**) All HSHF male samples; (**F**) All HSHF female samples. The symbol of N1A is the 7-day control male samples, N1B is the 7-day control female samples, and N2A for 14-day, N3A for 21-day, N4A for 28-day, N5A for 56-day male samples, N2B for 14-day, N3B for 21-day, N4B for 28-day, N5B for 56-day female samples; M1A is the 7-day HSHF male samples, M1B is the 7-day HSHF female samples, and M2A for 14-day, M3A for 21-day, M4A for 28-day, M5A for 56-day male samples, M2B for 14-day, M3B for 21-day, M4B for 28-day, M5B for 56-day female samples. Data are presented as the means ± SD of 8 independent experiments. **p* <0.05 and ***p* <0.01 vs. the model group by one-way ANOVA, followed by the one-way ANOVA test.

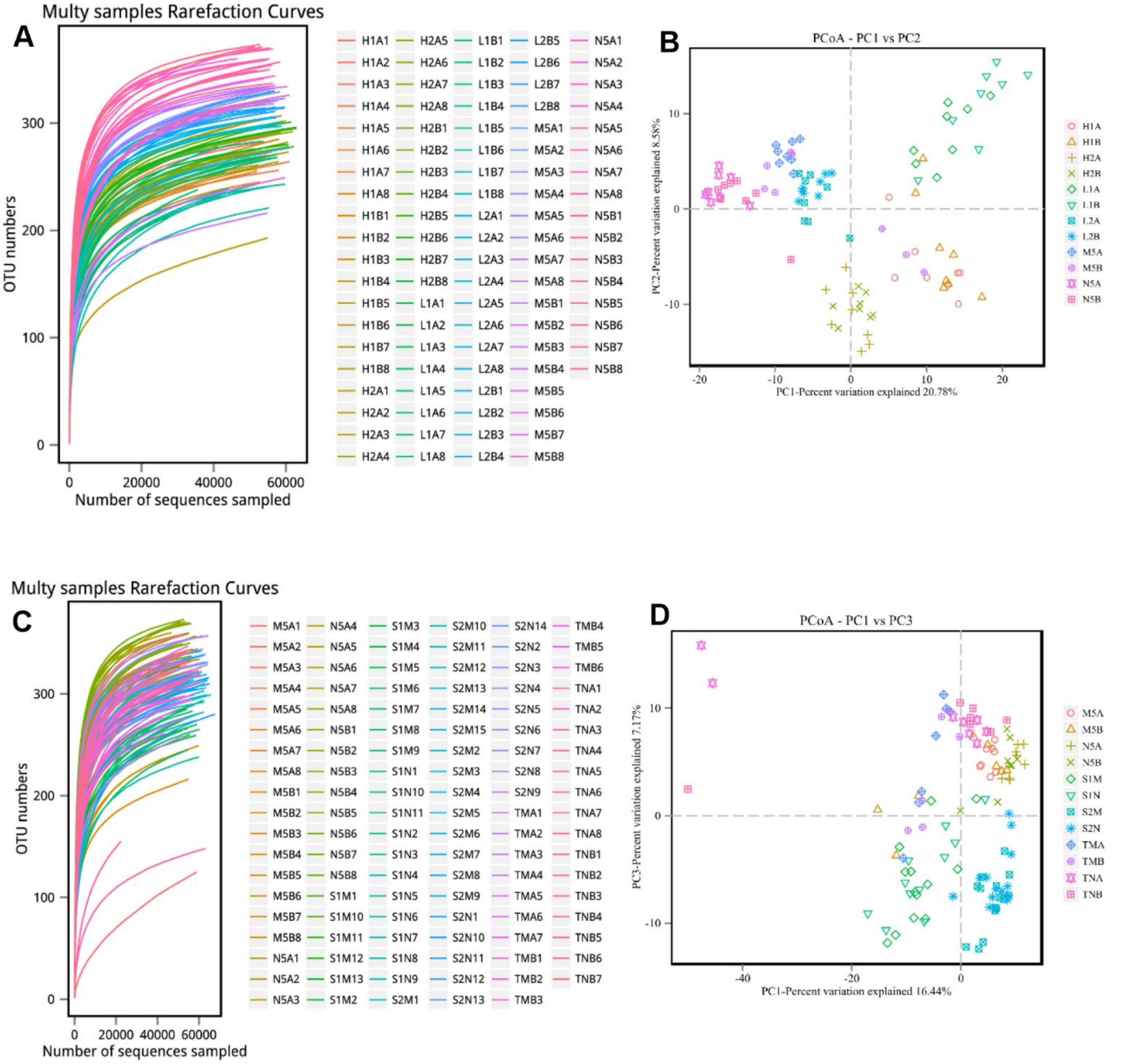

Supplementary Figure 2. Influences of maternal high sugar and fat diet on the alpha diversity and beta diversity of gut microbiota of offspring. (**A**) The alpha diversity of control, HSHF, *Hericium erinaceus*, *Ganoderma lucidum* treated samples at 28- and 56-day; (**B**) The beta diversity of control, HSHF, *Hericium erinaceus*, *Ganoderma lucidum* treated samples at 28- and 56-day; (**C**) The alpha diversity of control, HSHF, outdoor soil (T-) and from SAMP8 mice (S-) treated samples at 28- and 56-day; (**D**) The beta diversity of control, HSHF, outdoor soil (T-) and from SAMP8 mice (S-) treated samples at 28- and 56-day. The symbol of N1A is the 7-day control male samples, N1B is the 7-day control female samples, and N2A for 14-day, N3A for 21-day, N4A for 28-day, N5A for 56-day male samples, N2B for 14-day, N3B for 21-day, N4B for 28-day, N5B for 56-day female samples; M1A is the 7-day HSHF male samples, M1B is the 7-day HSHF female samples, and M2A for 14-day, M3A for 21-day, M4A for 28-day, M5A for 56-day male samples, M2B for 14-day, M3B for 21-day, M4B for 28-day, M5B for 56-day female samples. The symbol of N1 is the 28-day control samples, N2 is the 56-day control samples; M1 is the 28-day HSHF samples, M2 is the 56-day HSHF samples; *HE1* is for *Hericium erinaceus* treated 28-day samples, *HE2* is for *Hericium erinaceus* treated 56-day samples, *LZ1* is for *Ganoderma lucidum* treated 28-day samples, *LZ2* is for *Ganoderma lucidum* treated 56-day samples. Data are presented as the means ± SD of 8 independent experiments. $^{*}p$ <0.05 and $^{**}p$ <0.01 *vs*. the model group by one-way ANOVA test.

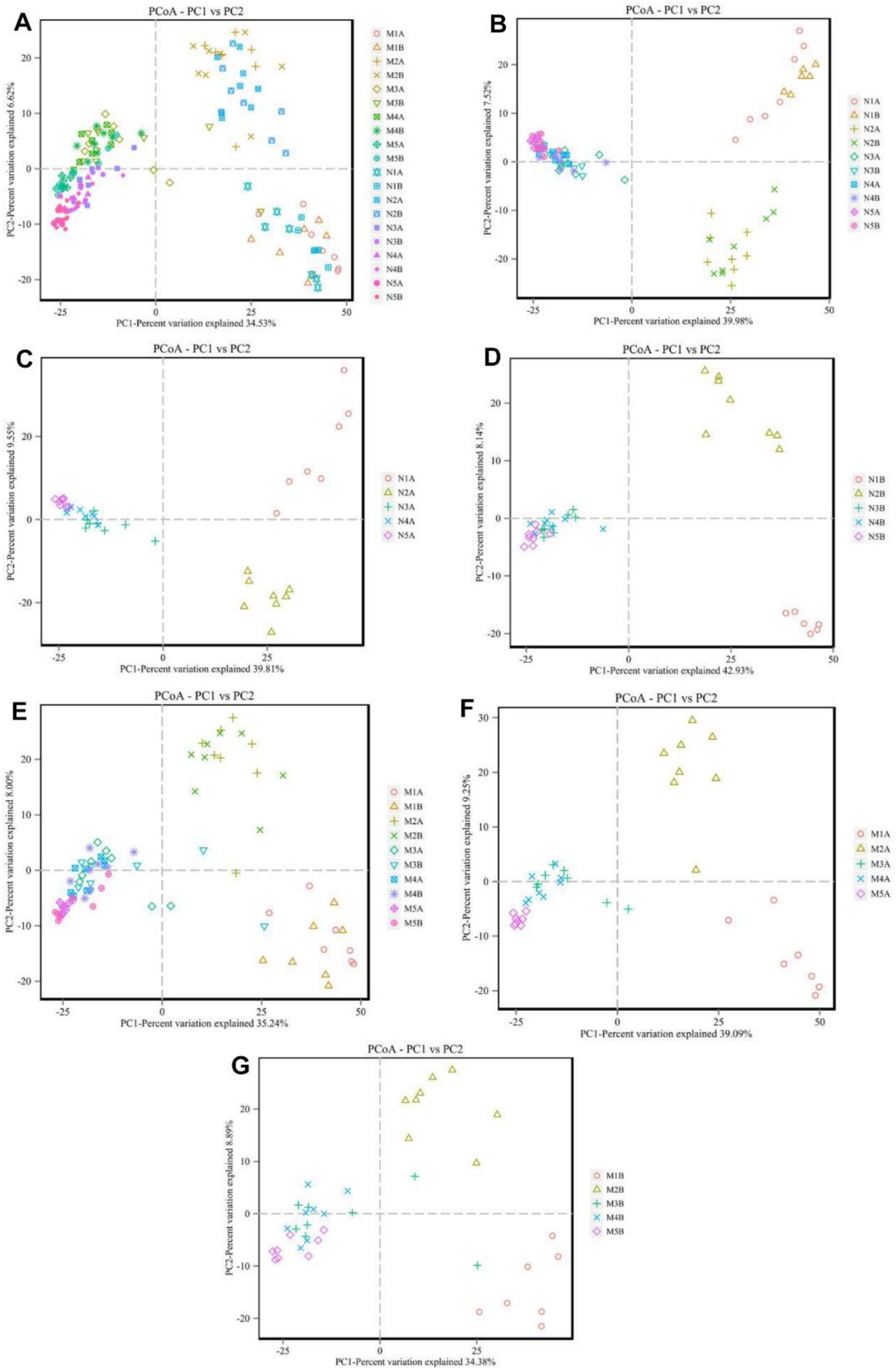

Supplementary Figure 3. Influences of maternal high sugar and fat diet on the gut microbiota of offspring. (**A**) All the samples; (**B**) All the control samples; (**C**) All the control male samples; (**D**) All the control female samples; (**E**) All the HSHF fed samples; (**F**) All HSHF male samples; (**G**) All HSHF female samples. The PCA analysis showed that the 7- (T1) and 14- (T2) days samples can successful distinguish of the 21- (T3), 28- (T4), 56- (T5) days samples, after 21 days, and the differences were decreased (Figure S2). The HSHF groups at different growth phase (7, 14, 21, 28, 56 days of age) showed the same variation tendency (Figure S2 and S4). The symbol of N1A is the 7-day control male samples, N1B is the 7-day control female samples, and N2A for 14-day, N3A for 21-day, N4A for 28-day, N5A for 56-day male samples, N2B for 14-day, N3B for 21-day, N4B for 28-day, N5B for 56-day female samples; M1A is the 7-day HSHF male samples, M1B is the 7-day HSHF female samples, and M2A for 14-day, M3A for 21-day, M4A for 28-day, M5A for 56-day male samples, M2B for 14-day, M3B for 21-day, M4B for 28-day, M5B for 56-day female samples. Data are presented as the means ± SD of 8 independent experiments. $^{*}p$ <0.05 and $^{**}p$ <0.01 *vs*. the model group by one-way ANOVA test.

A

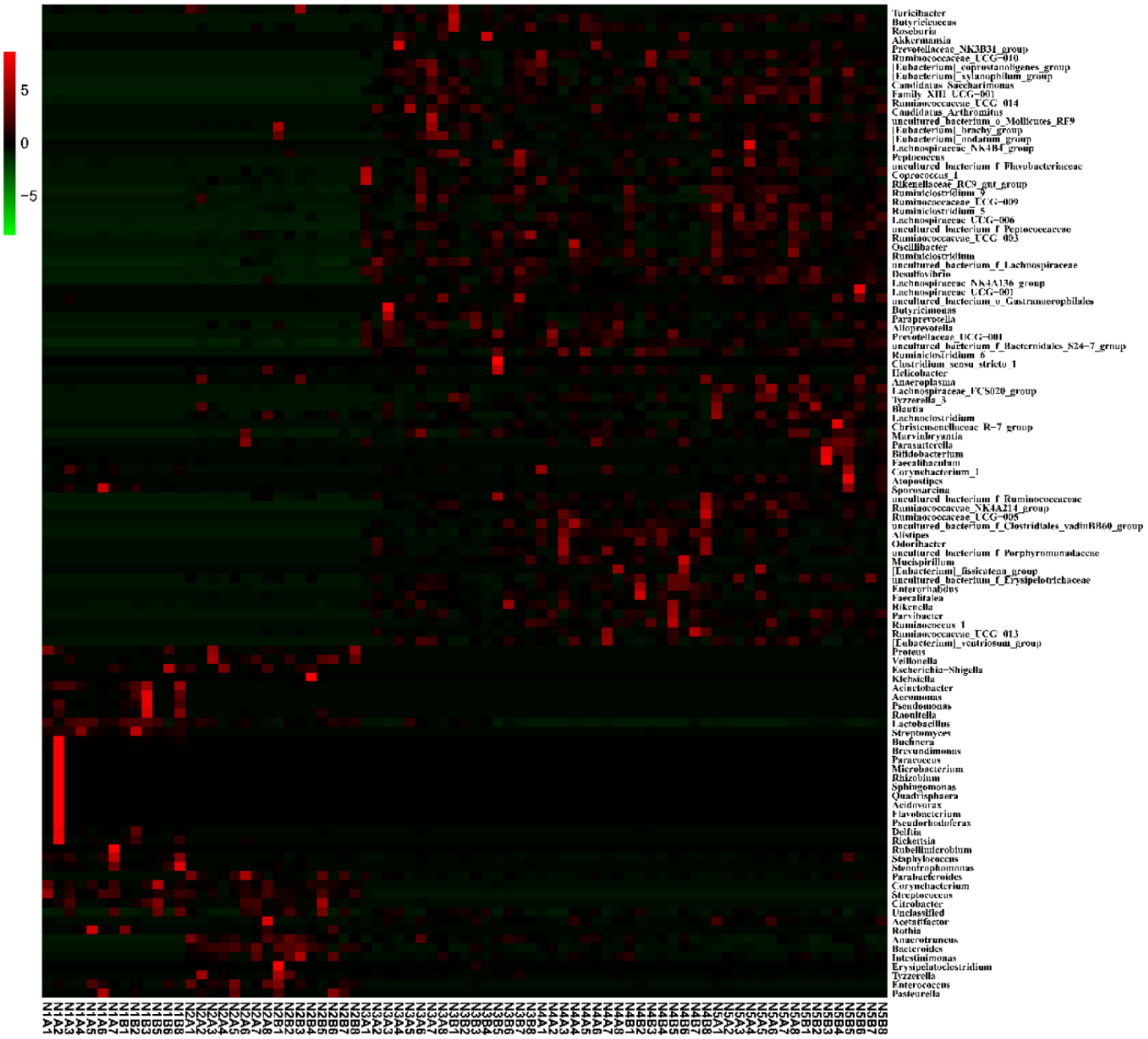
5
0
−5
Turicibacter
Butyricicoccus
Roseburia
Akkermansia
Prevotellaceae_NK3B31_group
Ruminococcaceae_UCG−010
[Eubacterium]_coprostanoligenes_group
[Eubacterium]_xylanophilum_group
Candidatus_Saccharimonas
Family_XIII_UCG−001
Ruminococcaceae_UCG_014
Candidatus_Arthromitus
uncultured_bacterium_o_Mollicutes_RF9
[Eubacterium]_brachy_group
[Eubacterium]_nodatum_group
Lachnospiraceae_NK4B4_group
Peptococcus
uncultured_bacterium_f_Flavobacteriaceae
Coprococcus_1
Rikenellaceae_RC9_gut_group
Ruminiclostridium_9
Ruminococcaceae_UCG−009
Ruminiclostridium_5
Lachnospiraceae_UCG−006
uncultured_bacterium_f_Peptococcaceae
Ruminococcaceae_UCG_003
Oscillibacter
Ruminiclostridium
uncultured_bacterium_f_Lachnospiraceae
Desulfovibrio
Lachnospiraceae_NK4A136_group
Lachnospiraceae_UCG−001
uncultured_bacterium_o_Gastranaerophilales
Butyricimonas
Paraprevotella
Alloprevotella
Prevotellaceae_UCG−001
uncultured_bacterium_f_Bacteroidales_S24−7_group
Ruminiclostridium_6
Clostridium_sensu_stricto_1
Helicobacter
Anaeroplasma
Lachnospiraceae_FCS020_group
Tyzzerella_3
Blautia
Lachnoclostridium
Christensenellaceae_R−7_group
Marvinbryantia
Parasutterella
Bifidobacterium
Faecalibaculum
Corynebacterium_1
Atopostipes
Sporosarcina
uncultured_bacterium_f_Ruminococcaceae
Ruminococcaceae_NK4A214_group
Ruminococcaceae_UCG−005
uncultured_bacterium_f_Clostridiales_vadinBB60_group
Alistipes
Odoribacter
uncultured_bacterium_f_Porphyromonadaceae
Mucispirillum
[Eubacterium]_fissicatena_group
uncultured_bacterium_f_Erysipelotrichaceae
Enterorhabdus
Faecalitalea
Rikenella
Parvibacter
Ruminococcus_1
Ruminococcaceae_UCG_013
[Eubacterium]_ventriosum_group
Proteus
Veillonella
Escherichia−Shigella
Klebsiella
Acinetobacter
Aeromonas
Pseudomonas
Raoultella
Lactobacillus
Streptomyces
Buchnera
Brevundimonas
Paracoccus
Microbacterium
Rhizobium
Sphingomonas
Quadrisphaera
Acidovorax
Flavobacterium
Pseudorhodoferax
Delftia
Rickettsia
Rubellimicrobium
Staphylococcus
Stenotrophomonas
Parabacteroides
Corynebacterium
Streptococcus
Citrobacter
Unclassified
Acetatifactor
Rothia
Anaerotruncus
Bacteroides
Intestinimonas
Erysipelatoclostridium
Tyzzerella
Enterococcus
Pasteurella

B

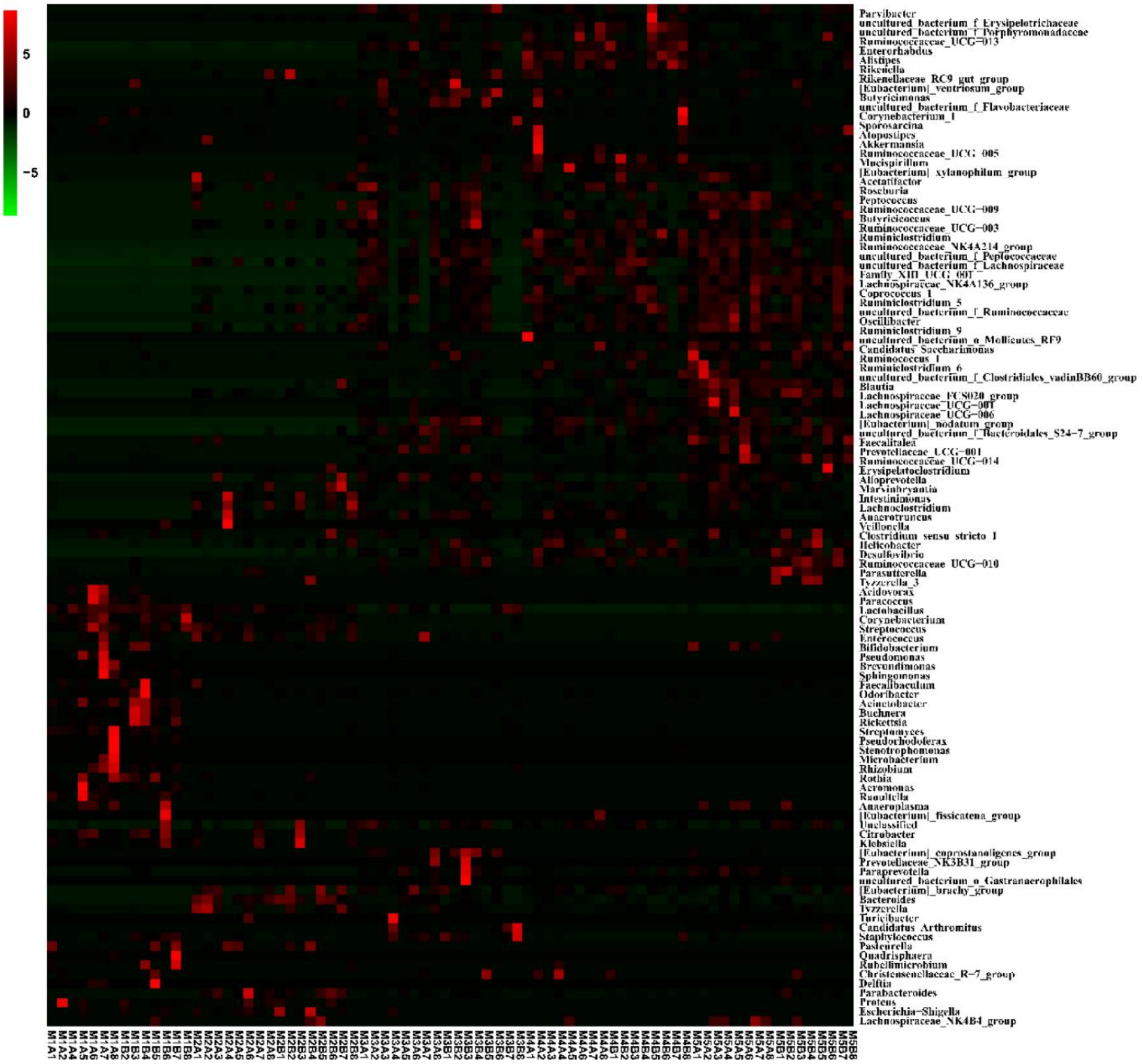
5
0
−5
Parvibacter
uncultured_bacterium_f_Erysipelotrichaceae
uncultured_bacterium_f_Porphyromonadaceae
Ruminococcaceae_UCG−013
Enterorhabdus
Alistipes
Rikenella
Rikenellaceae_RC9_gut_group
[Eubacterium]_ventriosum_group
Butyricimonas
uncultured_bacterium_f_Flavobacteriaceae
Corynebacterium_1
Sporosarcina
Atopostipes
Akkermansia
Ruminococcaceae_UCG−005
Mucispirillum
[Eubacterium]_xylanophilum_group
Acetatifactor
Roseburia
Peptococcus
Ruminococcaceae_UCG−009
Butyricicoccus
Ruminococcaceae_UCG−003
Ruminiclostridium
Ruminococcaceae_NK4A214_group
uncultured_bacterium_f_Peptococcaceae
uncultured_bacterium_f_Lachnospiraceae
Family_XIII_UCG−001
Lachnospiraceae_NK4A136_group
Coprococcus_1
Ruminiclostridium_5
uncultured_bacterium_f_Ruminococcaceae
Oscillibacter
Ruminiclostridium_9
uncultured_bacterium_o_Mollicutes_RF9
Candidatus_Saccharimonas
Ruminococcus_1
Ruminiclostridium_6
uncultured_bacterium_f_Clostridiales_vadinBB60_group
Blautia
Lachnospiraceae_FCS020_group
Lachnospiraceae_UCG−001
Lachnospiraceae_UCG−006
[Eubacterium]_nodatum_group
uncultured_bacterium_f_Bacteroidales_S24−7_group
Faecalitalea
Prevotellaceae_UCG−001
Ruminococcaceae_UCG−014
Erysipelatoclostridium
Alloprevotella
Marvinbryantia
Intestinimonas
Lachnoclostridium
Anaerotruncus
Veillonella
Clostridium_sensu_stricto_1
Helicobacter
Desulfovibrio
Ruminococcaceae_UCG−010
Parasutterella
Tyzzerella_3
Acidovorax
Paracoccus
Lactobacillus
Corynebacterium
Streptococcus
Enterococcus
Bifidobacterium
Pseudomonas
Brevundimonas
Sphingomonas
Faecalibaculum
Odoribacter
Acinetobacter
Buchnera
Rickettsia
Streptomyces
Pseudorhodoferax
Stenotrophomonas
Microbacterium
Rhizobium
Rothia
Aeromonas
Raoultella
Anaeroplasma
[Eubacterium]_fissicatena_group
Unclassified
Citrobacter
Klebsiella
[Eubacterium]_coprostanoligenes_group
Prevotellaceae_NK3B31_group
Paraprevotella
uncultured_bacterium_o_Gastranaerophilales
[Eubacterium]_brachy_group
Bacteroides
Tyzzerella
Turicibacter
Candidatus_Arthromitus
Staphylococcus
Pasteurella
Quadrisphaera
Rubellimicrobium
Christensenellaceae_R−7_group
Delftia
Parabacteroides
Proteus
Escherichia−Shigella
Lachnospiraceae_NK4B4_group

C

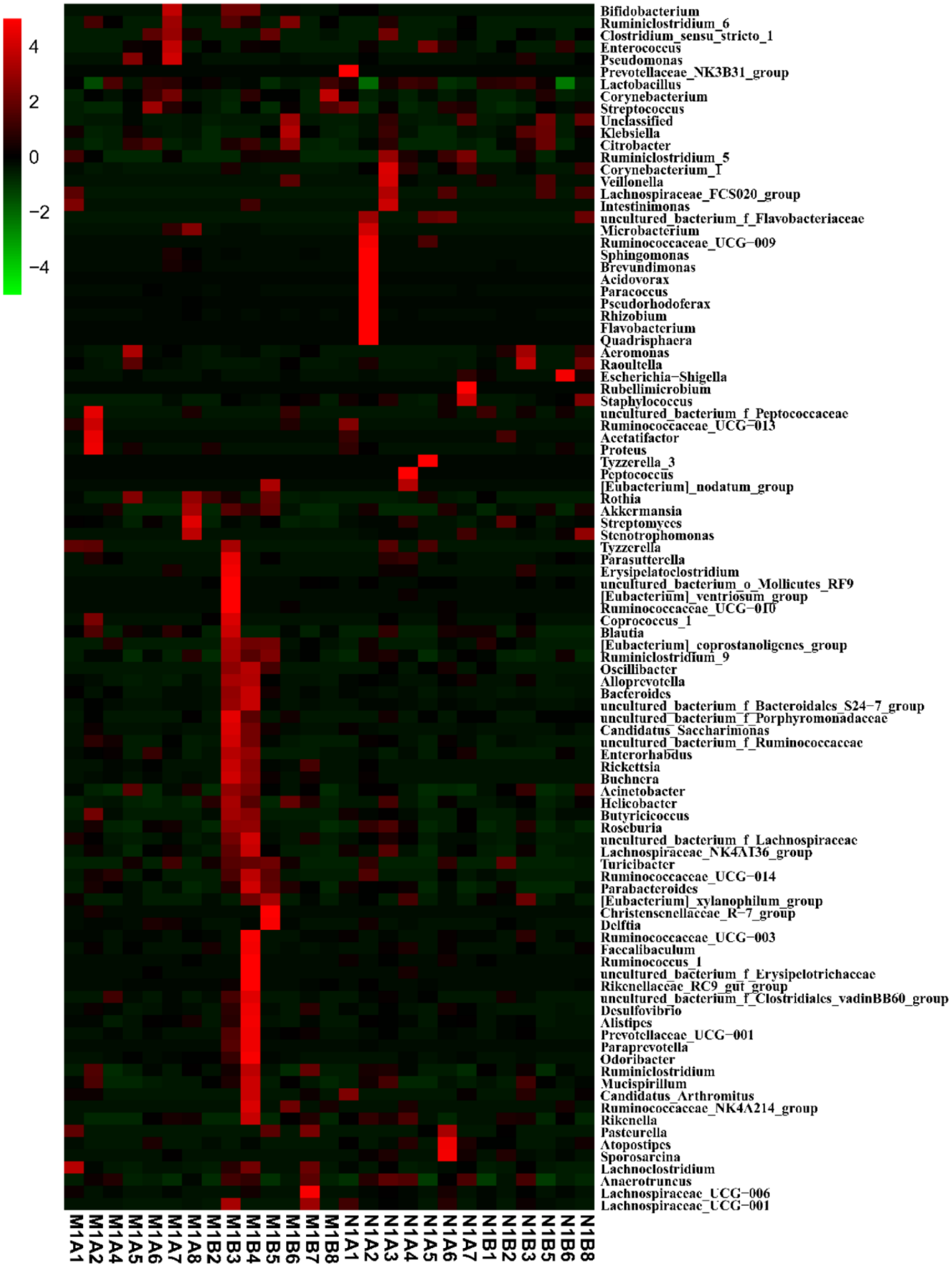
4
2
0
−2
−4
Bifidobacterium
Ruminiclostridium_6
Clostridium_sensu_stricto_1
Enterococcus
Pseudomonas
Prevotellaceae_NK3B31_group
Lactobacillus
Corynebacterium
Streptococcus
Unclassified
Klebsiella
Citrobacter
Ruminiclostridium_5
Corynebacterium_1
Veillonella
Lachnospiraceae_FCS020_group
Intestinimonas
uncultured_bacterium_f_Flavobacteriaceae
Microbacterium
Ruminococcaceae_UCG−009
Sphingomonas
Brevundimonas
Acidovorax
Paracoccus
Pseudorhodoferax
Rhizobium
Flavobacterium
Quadrisphaera
Aeromonas
Raoultella
Escherichia−Shigella
Rubellimicrobium
Staphylococcus
uncultured_bacterium_f_Peptococcaceae
Ruminococcaceae_UCG−013
Acetatifactor
Proteus
Tyzzerella_3
Peptococcus
[Eubacterium]_nodatum_group
Rothia
Akkermansia
Streptomyces
Stenotrophomonas
Tyzzerella
Parasutterella
Erysipelatoclostridium
uncultured_bacterium_o_Mollicutes_RF9
[Eubacterium]_ventriosum_group
Ruminococcaceae_UCG−010
Coprococcus_1
Blautia
[Eubacterium]_coprostanoligenes_group
Ruminiclostridium_9
Oscillibacter
Alloprevotella
Bacteroides
uncultured_bacterium_f_Bacteroidales_S24−7_group
uncultured_bacterium_f_Porphyromonadaceae
Candidatus_Saccharimonas
uncultured_bacterium_f_Ruminococcaceae
Enterorhabdus
Rickettsia
Buchnera
Acinetobacter
Helicobacter
Butyricicoccus
Roseburia
uncultured_bacterium_f_Lachnospiraceae
Lachnospiraceae_NK4A136_group
Turicibacter
Ruminococcaceae_UCG−014
Parabacteroides
[Eubacterium]_xylanophilum_group
Christensenellaceae_R−7_group
Delftia
Ruminococcaceae_UCG−003
Faecalibaculum
Ruminococcus_1
uncultured_bacterium_f_Erysipelotrichaceae
Rikenellaceae_RC9_gut_group
uncultured_bacterium_f_Clostridiales_vadinBB60_group
Desulfovibrio
Alistipes
Prevotellaceae_UCG−001
Paraprevotella
Odoribacter
Ruminiclostridium
Mucispirillum
Candidatus_Arthromitus
Ruminococcaceae_NK4A214_group
Rikenella
Pasteurella
Atopostipes
Sporosarcina
Lachnoclostridium
Anaerotruncus
Lachnospiraceae_UCG−006
Lachnospiraceae_UCG−001
M1A1
M1A2
M1A4
M1A5
M1A6
M1A7
M1A8
M1B2
M1B3
M1B4
M1B5
M1B6
M1B7
M1B8
N1A1
N1A2
N1A3
N1A4
N1A5
N1A6
N1A7
N1B1
N1B2
N1B3
N1B5
N1B6
N1B8

D

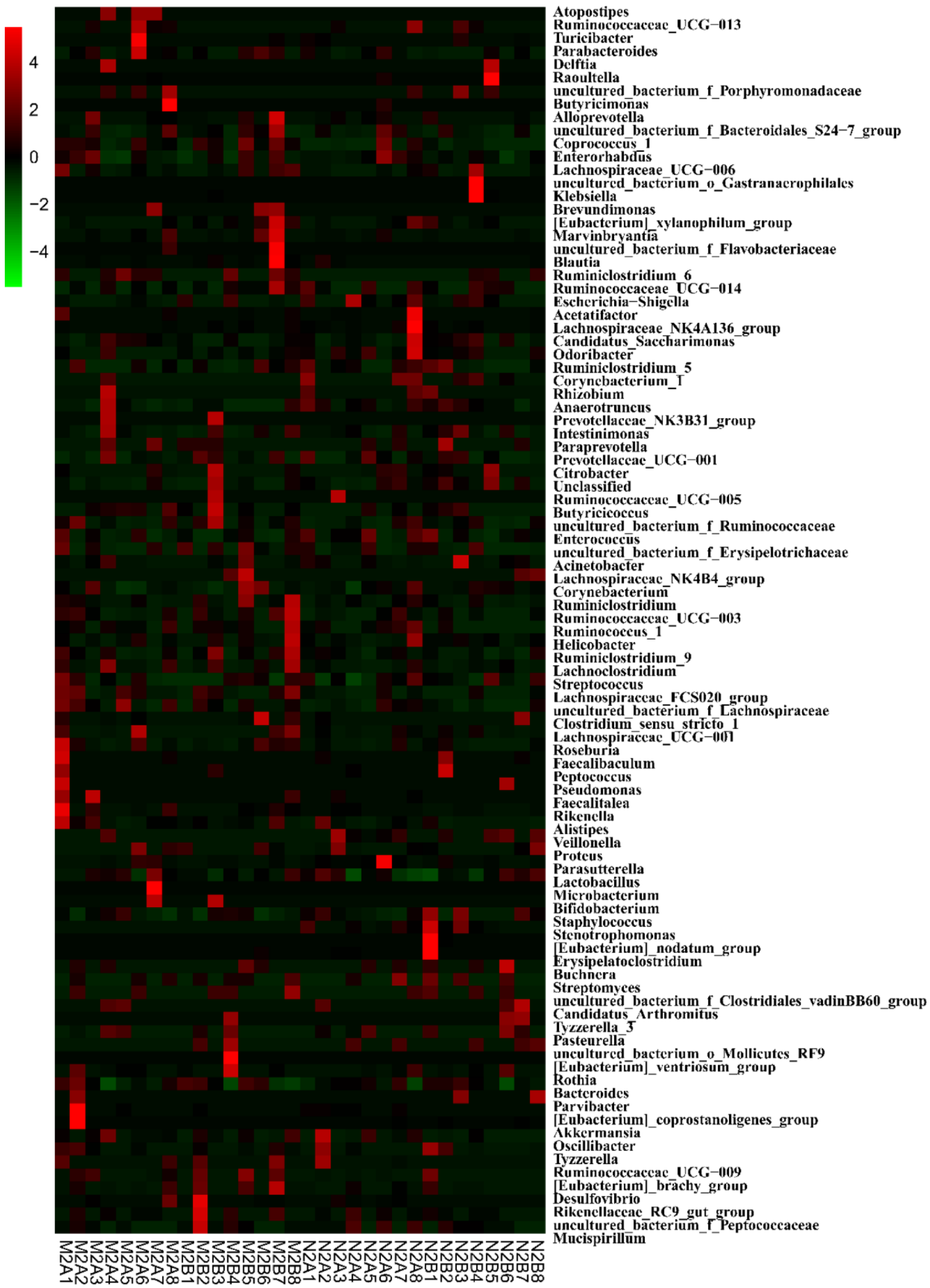
4
2
0
−2
−4
Atopostipes
Ruminococcaceae_UCG−013
Turicibacter
Parabacteroides
Delftia
Raoultella
uncultured_bacterium_f_Porphyromonadaceae
Butyricimonas
Alloprevotella
uncultured_bacterium_f_Bacteroidales_S24−7_group
Coprococcus_1
Enterorhabdus
Lachnospiraceae_UCG−006
uncultured_bacterium_o_Gastranaerophilales
Klebsiella
Brevundimonas
[Eubacterium]_xylanophilum_group
Marvinbryantia
uncultured_bacterium_f_Flavobacteriaceae
Blautia
Ruminiclostridium_6
Ruminococcaceae_UCG−014
Escherichia−Shigella
Acetatifactor
Lachnospiraceae_NK4A136_group
Candidatus_Saccharimonas
Odoribacter
Ruminiclostridium_5
Corynebacterium_1
Rhizobium
Anaerotruncus
Prevotellaceae_NK3B31_group
Intestinimonas
Paraprevotella
Prevotellaceae_UCG−001
Citrobacter
Unclassified
Ruminococcaceae_UCG−005
Butyricicoccus
uncultured_bacterium_f_Ruminococcaceae
Enterococcus
uncultured_bacterium_f_Erysipelotrichaceae
Acinetobacter
Lachnospiraceae_NK4B4_group
Corynebacterium
Ruminiclostridium
Ruminococcaceae_UCG−003
Ruminococcus_1
Helicobacter
Ruminiclostridium_9
Lachnoclostridium
Streptococcus
Lachnospiraceae_FCS020_group
uncultured_bacterium_f_Lachnospiraceae
Clostridium_sensu_stricto_1
Lachnospiraceae_UCG−001
Roseburia
Faecalibaculum
Peptococcus
Pseudomonas
Faecalitalea
Rikenella
Alistipes
Veillonella
Proteus
Parasutterella
Lactobacillus
Microbacterium
Bifidobacterium
Staphylococcus
Stenotrophomonas
[Eubacterium]_nodatum_group
Erysipelatoclostridium
Buchnera
Streptomyces
uncultured_bacterium_f_Clostridiales_vadinBB60_group
Candidatus_Arthromitus
Tyzzerella_3
Pasteurella
uncultured_bacterium_o_Mollicutes_RF9
[Eubacterium]_ventriosum_group
Rothia
Bacteroides
Parvibacter
[Eubacterium]_coprostanoligenes_group
Akkermansia
Oscillibacter
Tyzzerella
Ruminococcaceae_UCG−009
[Eubacterium]_brachy_group
Desulfovibrio
Rikenellaceae_RC9_gut_group
uncultured_bacterium_f_Peptococcaceae
Mucispirillum
M2A1
M2A2
M2A3
M2A4
M2A5
M2A6
M2A7
M2A8
M2B1
M2B2
M2B3
M2B4
M2B5
M2B6
M2B7
M2B8
N2A1
N2A2
N2A3
N2A4
N2A5
N2A6
N2A7
N2A8
N2B1
N2B2
N2B3
N2B4
N2B5
N2B6
N2B7
N2B8

E

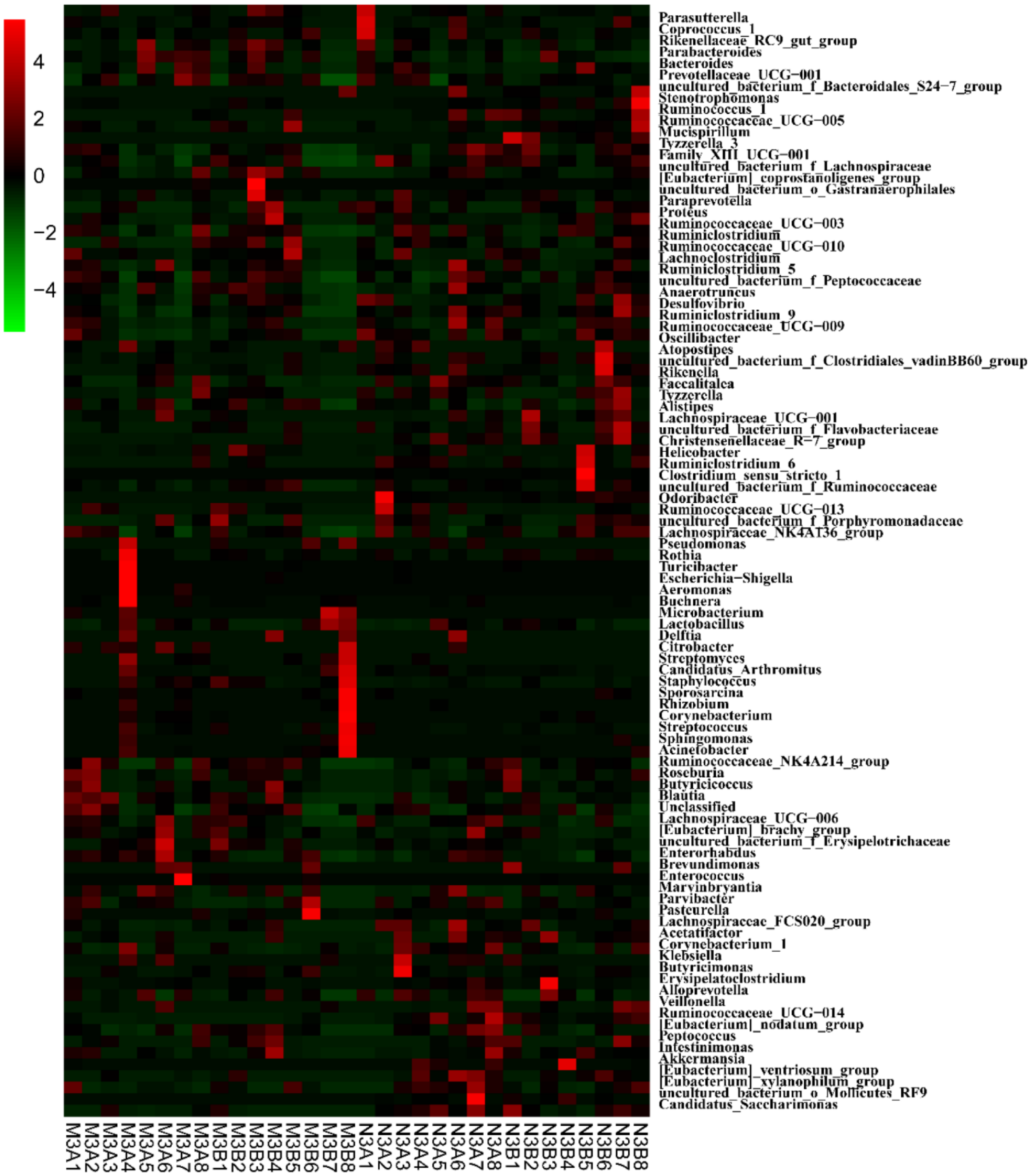
4
2
0
−2
−4
Parasutterella
Coprococcus_1
Rikenellaceae_RC9_gut_group
Parabacteroides
Bacteroides
Prevotellaceae_UCG−001
uncultured_bacterium_f_Bacteroidales_S24−7_group
Stenotrophomonas
Ruminococcus_1
Ruminococcaceae_UCG−005
Mucispirillum
Tyzzerella_3
Family_XIII_UCG−001
uncultured_bacterium_f_Lachnospiraceae
[Eubacterium]_coprostanoligenes_group
uncultured_bacterium_o_Gastranaerophilales
Paraprevotella
Proteus
Ruminococcaceae_UCG−003
Ruminiclostridium
Ruminococcaceae_UCG−010
Lachnoclostridium
Ruminiclostridium_5
uncultured_bacterium_f_Peptococcaceae
Anaerotruncus
Desulfovibrio
Ruminiclostridium_9
Ruminococcaceae_UCG−009
Oscillibacter
Atopostipes
uncultured_bacterium_f_Clostridiales_vadinBB60_group
Rikenella
Faecalitalea
Tyzzerella
Alistipes
Lachnospiraceae_UCG−001
uncultured_bacterium_f_Flavobacteriaceae
Christensenellaceae_R−7_group
Helicobacter
Ruminiclostridium_6
Clostridium_sensu_stricto_1
uncultured_bacterium_f_Ruminococcaceae
Odoribacter
Ruminococcaceae_UCG−013
uncultured_bacterium_f_Porphyromonadaceae
Lachnospiraceae_NK4A136_group
Pseudomonas
Rothia
Turicibacter
Escherichia−Shigella
Aeromonas
Buchnera
Microbacterium
Lactobacillus
Delftia
Citrobacter
Streptomyces
Candidatus_Arthromitus
Staphylococcus
Sporosarcina
Rhizobium
Corynebacterium
Streptococcus
Sphingomonas
Acinetobacter
Ruminococcaceae_NK4A214_group
Roseburia
Butyricicoccus
Blautia
Unclassified
Lachnospiraceae_UCG−006
[Eubacterium]_brachy_group
uncultured_bacterium_f_Erysipelotrichaceae
Enterorhabdus
Brevundimonas
Enterococcus
Marvinbryantia
Parvibacter
Pasteurella
Lachnospiraceae_FCS020_group
Acetatifactor
Corynebacterium_1
Klebsiella
Butyricimonas
Erysipelatoclostridium
Alloprevotella
Veillonella
Ruminococcaceae_UCG−014
[Eubacterium]_nodatum_group
Peptococcus
Intestinimonas
Akkermansia
[Eubacterium]_ventriosum_group
[Eubacterium]_xylanophilum_group
uncultured_bacterium_o_Mollicutes_RF9
Candidatus_Saccharimonas
M3A1
M3A2
M3A3
M3A4
M3A5
M3A6
M3A7
M3A8
M3B1
M3B2
M3B3
M3B4
M3B5
M3B6
M3B7
M3B8
N3A1
N3A2
N3A3
N3A4
N3A5
N3A6
N3A7
N3A8
N3B1
N3B2
N3B3
N3B4
N3B5
N3B6
N3B7
N3B8

F

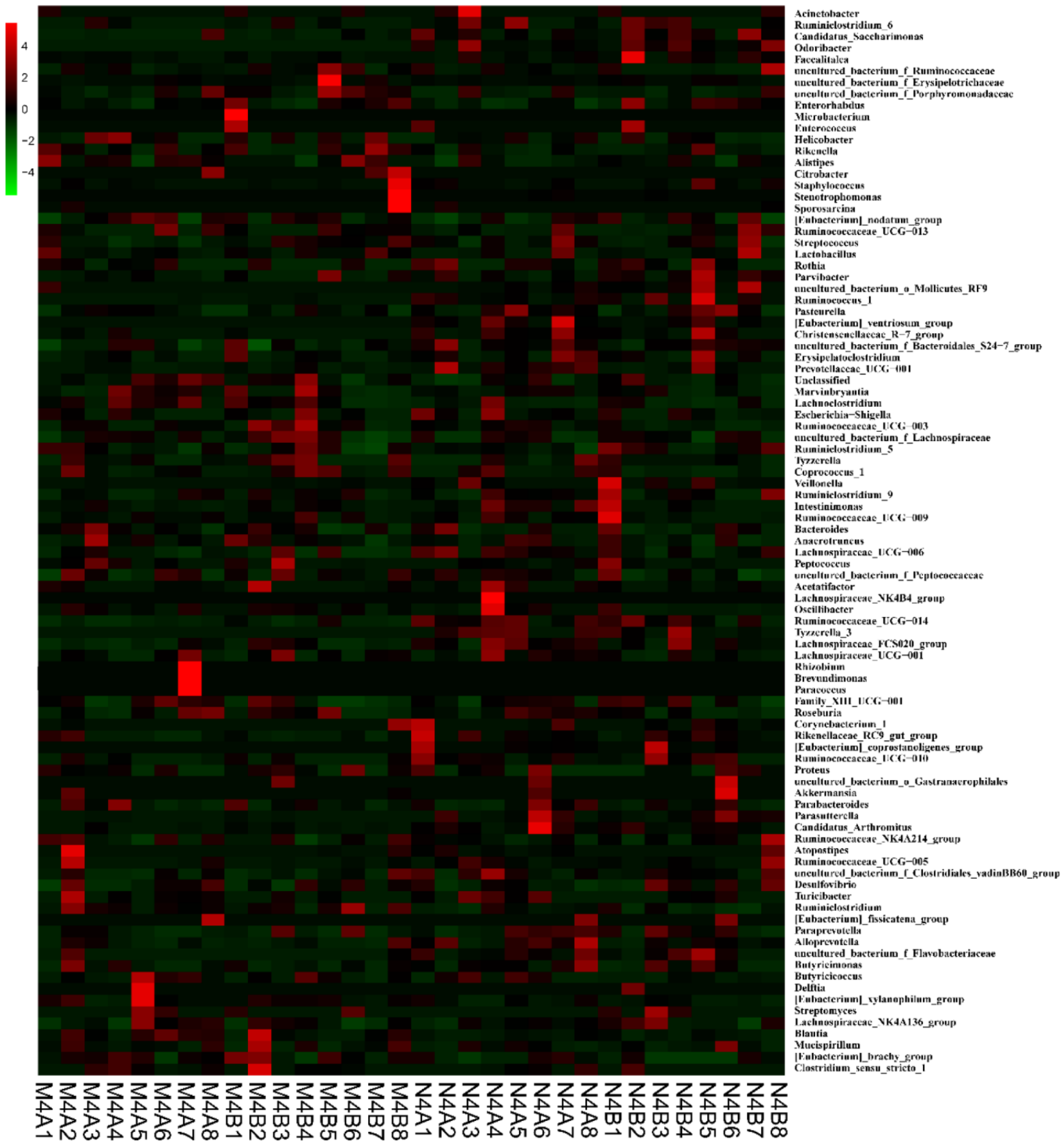
4
2
0
-2
-4
Acinetobacter
Ruminiclostridium_6
Candidatus_Saccharimonas
Odoribacter
Faecalitalea
uncultured_bacterium_f_Ruminococcaceae
uncultured_bacterium_f_Erysipelotrichaceae
uncultured_bacterium_f_Porphyromonadaceae
Enterorhabdus
Microbacterium
Enterococcus
Helicobacter
Rikenella
Alistipes
Citrobacter
Staphylococcus
Stenotrophomonas
Sporosarcina
[Eubacterium]_nodatum_group
Ruminococcaceae_UCG−013
Streptococcus
Lactobacillus
Rothia
Parvibacter
uncultured_bacterium_o_Mollicutes_RF9
Ruminococcus_1
Pasteurella
[Eubacterium]_ventriosum_group
Christensenellaceae_R−7_group
uncultured_bacterium_f_Bacteroidales_S24−7_group
Erysipelatoclostridium
Prevotellaceae_UCG−001
Unclassified
Marvinbryantia
Lachnoclostridium
Escherichia−Shigella
Ruminococcaceae_UCG−003
uncultured_bacterium_f_Lachnospiraceae
Ruminiclostridium_5
Tyzzerella
Coprococcus_1
Veillonella
Ruminiclostridium_9
Intestinimonas
Ruminococcaceae_UCG−009
Bacteroides
Anaerotruncus
Lachnospiraceae_UCG−006
Peptococcus
uncultured_bacterium_f_Peptococcaceae
Acetatifactor
Lachnospiraceae_NK4B4_group
Oscillibacter
Ruminococcaceae_UCG−014
Tyzzerella_3
Lachnospiraceae_FCS020_group
Lachnospiraceae_UCG−001
Rhizobium
Brevundimonas
Paracoccus
Family_XIII_UCG−001
Roseburia
Corynebacterium_1
Rikenellaceae_RC9_gut_group
[Eubacterium]_coprostanoligenes_group
Ruminococcaceae_UCG−010
Proteus
uncultured_bacterium_o_Gastranaerophilales
Akkermansia
Parabacteroides
Parasutterella
Candidatus_Arthromitus
Ruminococcaceae_NK4A214_group
Atopostipes
Ruminococcaceae_UCG−005
uncultured_bacterium_f_Clostridiales_vadinBB60_group
Desulfovibrio
Turicibacter
Ruminiclostridium
[Eubacterium]_fissicatena_group
Paraprevotella
Alloprevotella
uncultured_bacterium_f_Flavobacteriaceae
Butyricimonas
Butyricicoccus
Delftia
[Eubacterium]_xylanophilum_group
Streptomyces
Lachnospiraceae_NK4A136_group
Blautia
Mucispirillum
[Eubacterium]_brachy_group
Clostridium_sensu_stricto_1
M4A1
M4A2
M4A3
M4A4
M4A5
M4A6
M4A7
M4A8
M4B1
M4B2
M4B3
M4B4
M4B5
M4B6
M4B7
M4B8
N4A1
N4A2
N4A3
N4A4
N4A5
N4A6
N4A7
N4A8
N4B1
N4B2
N4B3
N4B4
N4B5
N4B6
N4B7
N4B8

G

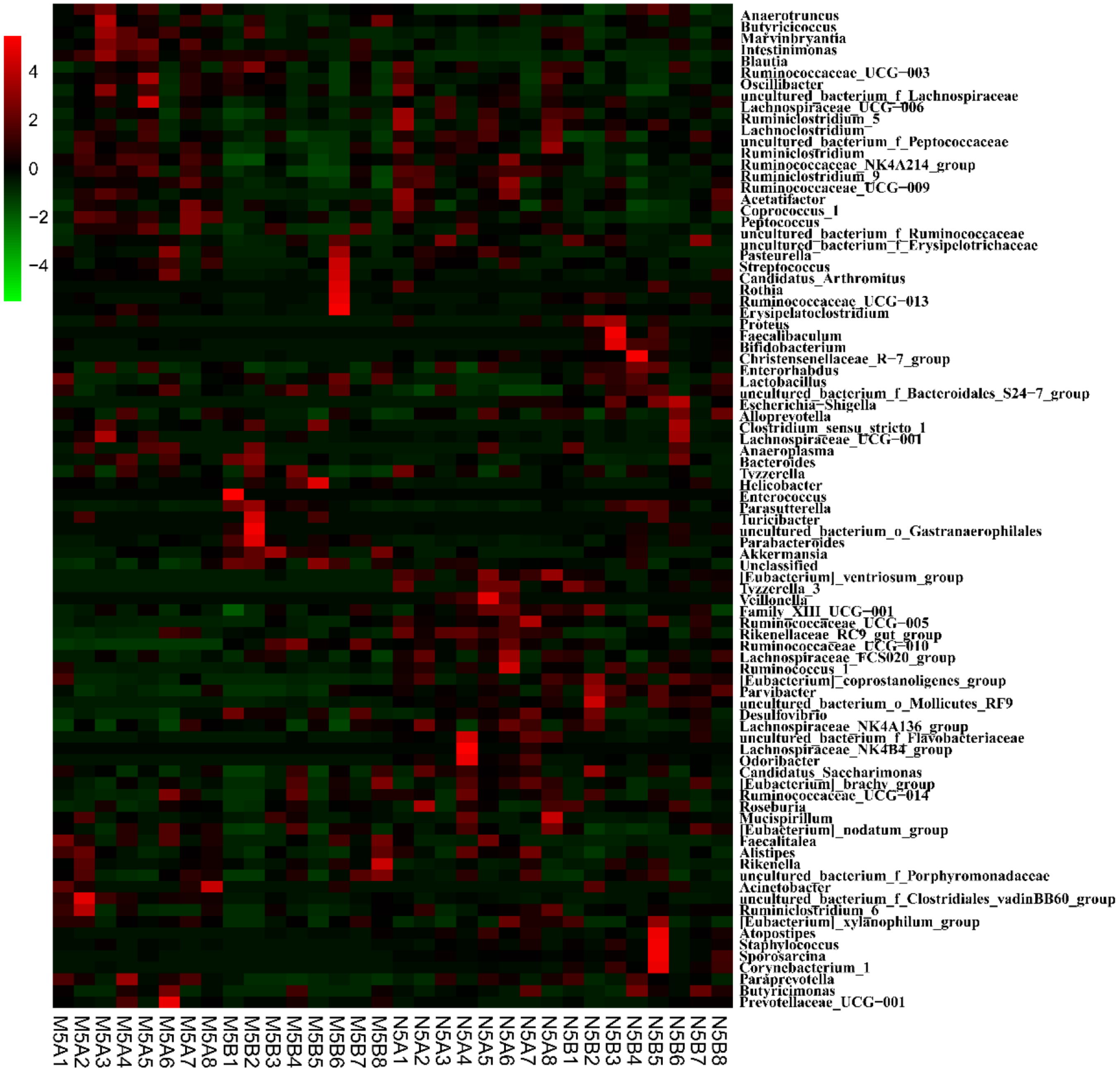

Supplementary Figure 4. Influences of maternal high sugar and fat diet on the genus of gut microbiota of offspring. (**A**) All the control samples; (**B**) All the HSHF fed samples; (**C**) All the samples at 7-day; (**D**) All the samples at 14-day; (**E**) All the samples at 21-day; (**F**) All the samples at 28-day; (**G**) All the samples at 56-day. The symbol of N1A is the 7-day control male samples, N1B is the 7-day control female samples, and N2A for 14-day, N3A for 21-day, N4A for 28-day, N5A for 56-day male samples, N2B for 14-day, N3B for 21-day, N4B for 28-day, N5B for 56-day female samples; M1A is the 7-day HSHF male samples, M1B is the 7-day HSHF female samples, and M2A for 14-day, M3A for 21-day, M4A for 28-day, M5A for 56-day male samples, M2B for 14-day, M3B for 21-day, M4B for 28-day, M5B for 56-day female samples. Data are presented as the means ± SD of 8 independent experiments. **p* <0.05 and ***p* <0.01 vs. the model group by one-way ANOVA, followed by the one-way ANOVA test.

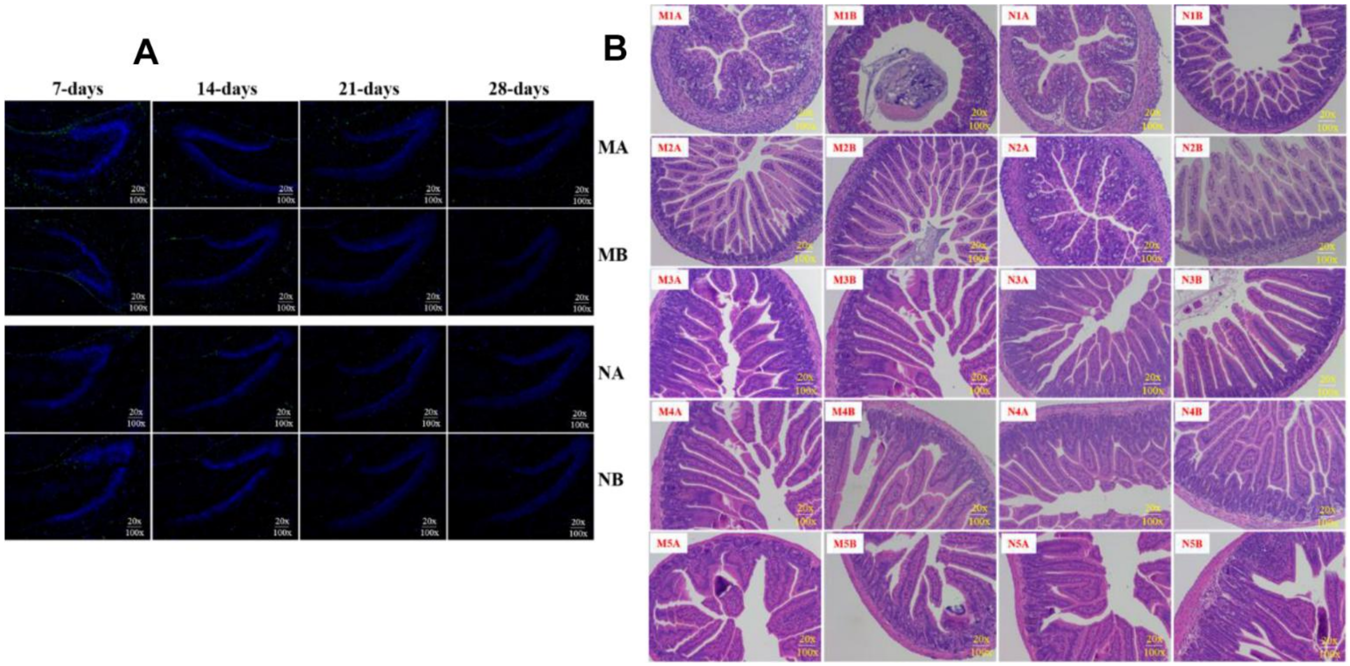

Supplementary Figure 5. The pathological structure of brain and small intestine. IBA1 for microglia (**A**); H&E staining showed the pathological structure of the small intestine (**B**). The symbol of N1A is the 7-day control male samples, N1B is the 7-day control female samples, and N2A for 14-day, N3A for 21-day, N4A for 28-day, N5A for 56-day male samples, N2B for 14-day, N3B for 21-day, N4B for 28-day, N5B for 56-day female samples; M1A is the 7-day HSHF male samples, M1B is the 7-day HSHF female samples, and M2A for 14-day, M3A for 21-day, M4A for 28-day, M5A for 56-day male samples, M2B for 14-day, M3B for 21-day, M4B for 28-day, M5B for 56-day female samples.

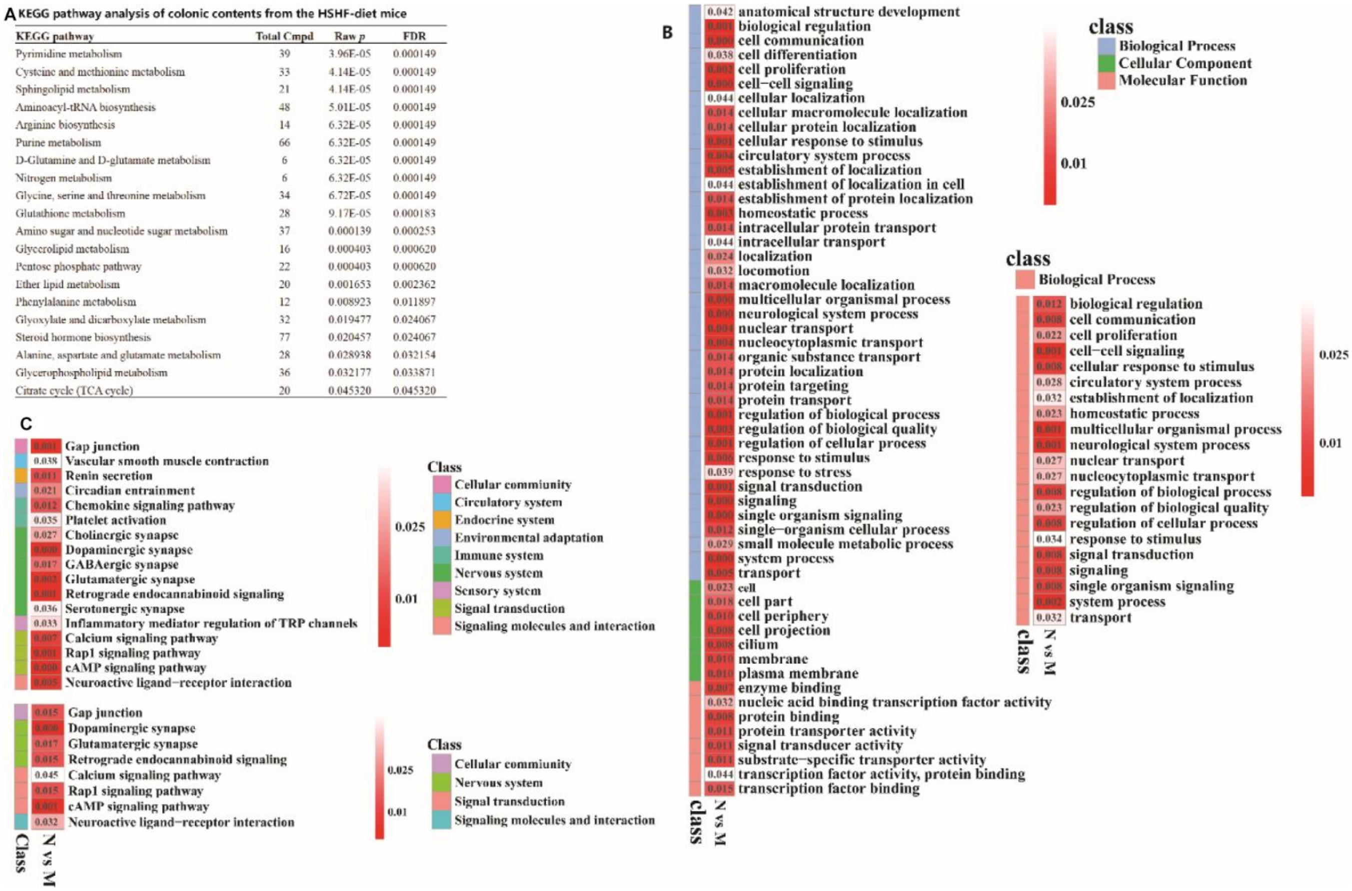

Supplementary Figure 6. Pathway analysis of the different metabolites in colonic contents from the HSHF-diet mice using MetaboAnalyst 4.0 (**A**); The cholinergic neurons and GABA neurons were increased by up-regulated the LHX8 in the HSHF diet offspring when they getting old (**B**, **C**). The KEGG analysis showed that the significant pathways including Dopaminergic synapse, cAMP signaling pathway, Gap junction, Rap1 signaling pathway, Retrograde endocannabinoid signaling, Glutamatergic synapse, Neuroactive ligand-receptor interaction, Calcium signaling pathway.

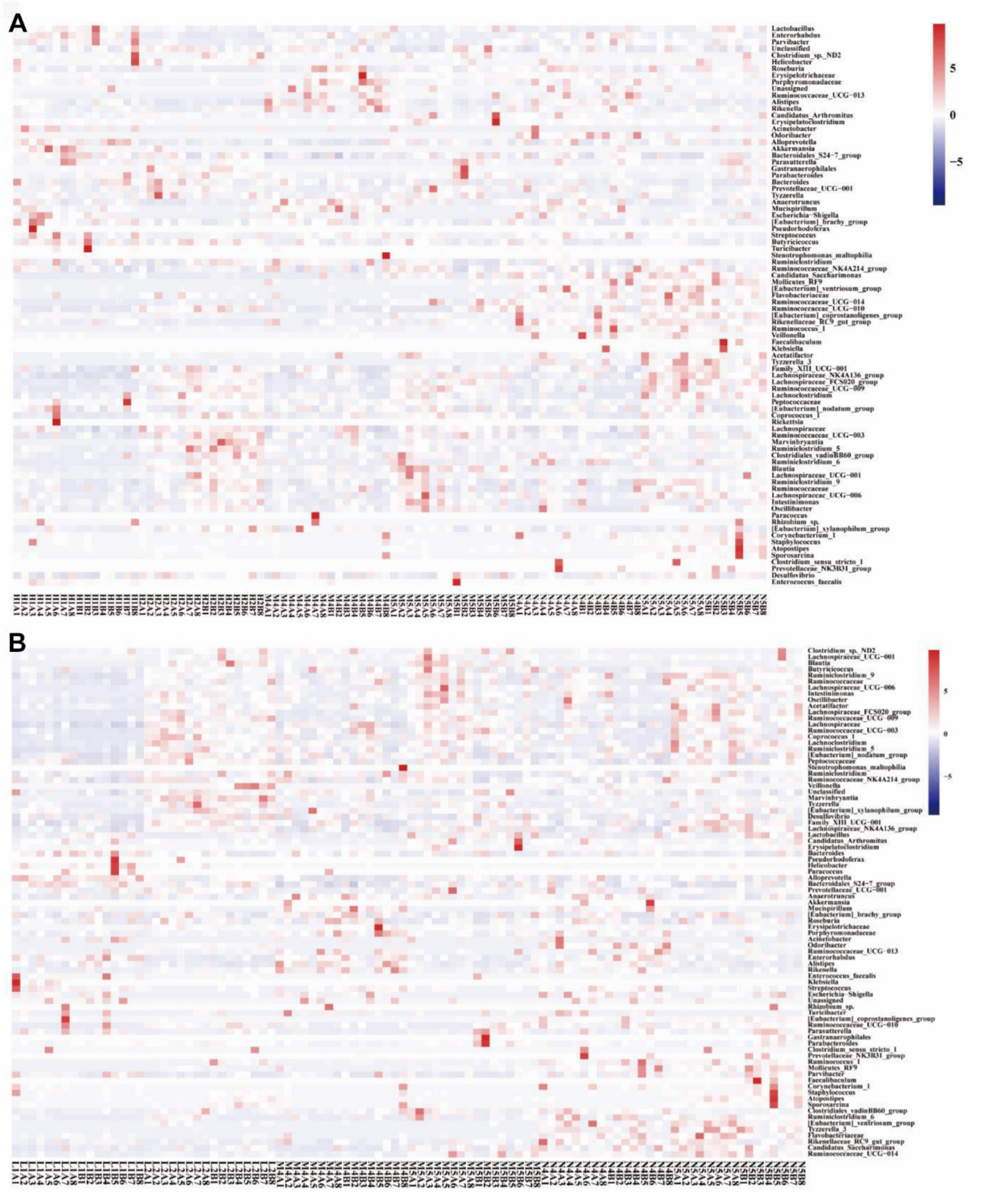

Supplementary Figure 7. Special maternal diet changes the gut microbiome trace. (**A**) The dietary supplement of *Hericium erinaceus* group (**A**); the dietary supplement of *Ganoderma lucidum* (**B**). The symbol of N1A is the 7-day control male samples, N1B is the 7-day control female samples, and N2A for 14-day, N3A for 21-day, N4A for 28-day, N5A for 56-day male samples, N2B for 14-day, N3B for 21-day, N4B for 28-day, N5B for 56-day female samples; M1A is the 7-day HSHF male samples, M1B is the 7-day HSHF female samples, and M2A for 14-day, M3A for 21-day, M4A for 28-day, M5A for 56-day male samples, M2B for 14-day, M3B for 21-day, M4B for 28-day, M5B for 56-day female samples. The symbol of N1 is the 28-day control samples, N2 is the 56-day control samples; M1 is the 28-day HSHF samples, M2 is the 56-day HSHF samples; *HE1* is for *Hericium erinaceus* treated 28-day samples, *HE2* is for *Hericium erinaceus* treated 56-day samples, *LZ1* is for *Ganoderma lucidum* treated 28-day samples, *LZ2* is for *Ganoderma lucidum* treated 56-day samples. Data are presented as the means ± SD of 8 independent experiments. $^{*}p$ <0.05 and $^{**}p$ <0.01 *vs*. the model group by one-way ANOVA, followed by the one-way ANOVA test.

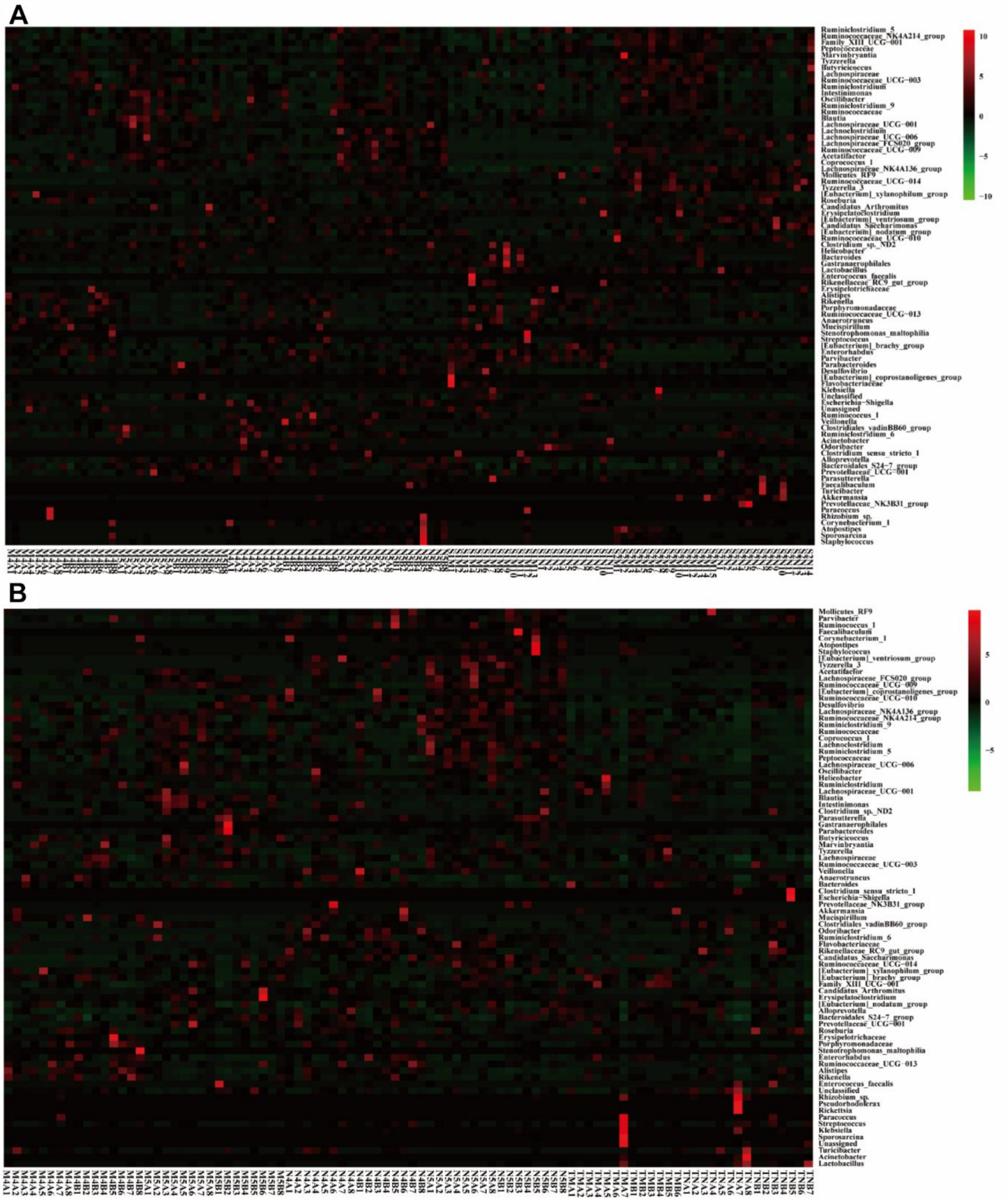

Supplementary Figure 8. Special maternal diet changes the gut microbiome trace. The exposure to different microbe in early life (microbe from the outdoor soil (T-, **A**) and SAMP8 mice (S-, **B**) also could change the inherent species abundance (Figure S3). The symbol of N1A is the 7-day control male samples, N1B is the 7-day control female samples, and N2A for 14-day, N3A for 21-day, N4A for 28-day, N5A for 56-day male samples, N2B for 14-day, N3B for 21-day, N4B for 28-day, N5B for 56-day female samples; M1A is the 7-day HSHF male samples, M1B is the 7-day HSHF female samples, and M2A for 14-day, M3A for 21-day, M4A for 28-day, M5A for 56-day male samples, M2B for 14-day, M3B for 21-day, M4B for 28-day, M5B for 56-day female samples. The symbol of N1 is the 28-day control samples, N2 is the 56-day control samples; M1 is the 28-day HSHF samples, M2 is the 56-day HSHF samples; *HE1* is for *Hericium erinaceus* treated 28-day samples, *HE2* is for *Hericium erinaceus* treated 56-day samples, *LZ1* is for *Ganoderma lucidum* treated 28-day samples, *LZ2* is for *Ganoderma lucidum* treated 56-day samples. Data are presented as the means ± SD of 8 independent experiments. $^{*}p$ <0.05 and $^{**}p$ <0.01 *vs*. the model group by one-way ANOVA, followed by the one-way ANOVA test.
