# Supplementary material for: Maternal high sugar and fat diet benefits offspring brain function via targeting on the gut–brain axis
Source: Aging (Albany NY). 2021 Mar 26;13(7):10240–74. doi: 10.18632/aging.202787 (PMC8064210; doi:10.18632/aging.202787)
Supplement: Supplementary Table 1 [file aging-13-202787-s002.pdf]

## SUPPLEMENTARY TABLE

**Supplementary Table 1. The different expressed mRNA in the offspring of whose mother fed HSHF diet.**

| Symbol | N_fpkm | M_fpkm | log2(FC) | *P*-value | FDR | Significant |
|---|---|---|---|---|---|---|
| Rxrg | 1.3000 | 8.5167 | 2.7308 | 2.61E-08 | 3.82E-05 | up |
| Rarb | 2.3933 | 6.8767 | 1.5945 | 5.05E-06 | 3.70E-03 | up |
| Kpna2 | 14.5300 | 4.6467 | -1.6530 | 3.77E-06 | 2.88E-03 | down |
| Slc17a8 | 0.2867 | 0.9700 | 2.1646 | 6.12E-05 | 3.33E-02 | up |
| Adora2a | 1.3200 | 17.6900 | 3.8129 | 1.26E-27 | 1.20E-23 | up |
| Rgs9 | 6.1533 | 42.7467 | 2.8179 | 1.25E-22 | 7.94E-19 | up |
| Drd1 | 1.2067 | 8.0733 | 2.7902 | 5.61E-08 | 7.63E-05 | up |
| Pde1b | 34.1267 | 92.4600 | 1.5092 | 9.75E-07 | 8.85E-04 | up |
| Adcy5 | 20.4867 | 48.9767 | 1.3243 | 1.07E-05 | 7.01E-03 | up |
| Cd4 | 0.2133 | 3.3833 | 3.8388 | 6.68E-10 | 1.41E-06 | up |
| Meis2 | 17.3900 | 41.5400 | 1.3304 | 1.01E-04 | 4.55E-02 | up |
| Pdyn | 2.6300 | 11.3500 | 2.1636 | 7.88E-07 | 7.90E-04 | up |
| Syt6 | 5.4200 | 13.7333 | 1.4156 | 2.84E-07 | 3.00E-04 | up |
| Ido1 | 0.0867 | 1.0033 | 3.5852 | 1.86E-06 | 1.48E-03 | up |
| Drd2 | 2.1800 | 15.8833 | 2.9403 | 1.97E-13 | 7.50E-10 | up |
| Rasgrp2 | 35.8433 | 70.9600 | 1.3913 | 9.38E-08 | 1.19E-04 | up |
| Asic4 | 2.7333 | 8.6967 | 1.7342 | 8.60E-06 | 5.85E-03 | up |
| Rasd2 | 14.8867 | 43.3400 | 1.6131 | 1.67E-06 | 1.38E-03 | up |
| Dsg1c | 0.0200 | 0.2133 | 3.4806 | 8.28E-06 | 5.84E-03 | up |
| Six3 | 0.2800 | 3.4967 | 3.7379 | 1.73E-07 | 2.06E-04 | up |
| Isl1 | 0.2533 | 2.5767 | 3.2344 | 1.49E-05 | 9.13E-03 | up |
| Penk | 97.9967 | 226.9467 | 1.2404 | 1.00E-04 | 4.55E-02 | up |
| Gpr6 | 0.5867 | 6.8467 | 3.5876 | 2.19E-07 | 2.45E-04 | up |
| Gng7 | 21.5200 | 71.1867 | 1.7225 | 8.33E-07 | 7.93E-04 | up |
| Slc35d3 | 0.6433 | 3.3900 | 2.4592 | 1.45E-06 | 1.25E-03 | up |
| Sh3rf2 | 0.1867 | 1.8233 | 3.6057 | 1.53E-10 | 4.15E-07 | up |
| Serpina9 | 0.7500 | 5.3200 | 2.9103 | 1.91E-09 | 3.30E-06 | up |
| Ppp1r1b | 71.8133 | 167.5233 | 1.3000 | 1.34E-05 | 8.53E-03 | up |
| Tac1 | 9.1000 | 51.9433 | 2.5360 | 4.38E-10 | 1.04E-06 | up |
| Gm10184 | 0.1733 | 10.7800 | 5.9345 | 9.93E-29 | 1.89E-24 | up |
| Gpr88 | 4.2200 | 53.4033 | 3.4044 | 9.59E-20 | 4.57E-16 | up |
| Syndig1l | 6.5100 | 15.8800 | 1.3751 | 8.42E-05 | 4.11E-02 | up |
| Lrrc10b | 2.2600 | 16.3133 | 2.9145 | 7.49E-05 | 3.96E-02 | up |
| Fam205a3 | 1.6233 | 4.7567 | 1.6366 | 7.94E-05 | 3.98E-02 | up |
| Fam205a2 | 1.6233 | 4.7567 | 1.6366 | 7.94E-05 | 3.98E-02 | up |
| Lhx8 | 0.0010 | 1.7533 | 8.5055 | 1.08E-09 | 2.07E-06 | up |
| TCONS_00007919 | 0.4000 | 2.4333 | 2.6665 | 1.31E-08 | 2.08E-05 | up |
| TCONS_00015012 | 0.0633 | 0.7033 | 3.4096 | 1.82E-05 | 1.05E-02 | up |
| TCONS_00085226 | 1.3800 | 0.0533 | -4.5407 | 1.03E-10 | 3.28E-07 | down |
| TCONS_00104776 | 49.1267 | 8.1100 | -2.5287 | 1.75E-05 | 1.04E-02 | down |
| TCONS_00139120 | 0.7967 | 0.0533 | -3.8060 | 1.03E-04 | 4.55E-02 | down |
